# Double-layered N-S1 protein nanoparticle immunization elicits robust cellular immune and broad antibody responses against SARS-CoV-2

**DOI:** 10.1186/s12951-024-02293-y

**Published:** 2024-01-30

**Authors:** Ruiqi Li, Zejie Chang, Hongliang Liu, Yanan Wang, Minghui Li, Yilan Chen, Lu Fan, Siqiao Wang, Xueke Sun, Siyuan Liu, Anchun Cheng, Peiyang Ding, Gaiping Zhang

**Affiliations:** 1 College of Veterinary Medicine, Sichuan Agricultural University, Chengdu, 611130 China; 2 School of Advanced Agricultural Sciences , Peking University, Beijing, 100080 China; 3 Longhu Laboratory, Zhengzhou, 450046 China; 4 Henan Provincial Key Laboratory of Animal Immunology, Henan Academy of Agricultural Sciences, Zhengzhou, 450002 China; 5 College of Animal Medicine, Henan Agricultural University, Zhengzhou, 450046 China; 6 School of Life Sciences , Zhengzhou University, Zhengzhou, 450001 China

**Keywords:** COVID-19, Coronavirus, SARS-CoV-2, Nanoparticle, Subunit vaccine, Variants

## Abstract

**Background:**

The COVID-19 pandemic is a persistent global threat to public health. As for the emerging variants of SARS-CoV-2, it is necessary to develop vaccines that can induce broader immune responses, particularly vaccines with weak cellular immunity.

**Methods:**

In this study, we generated a double-layered N-S1 protein nanoparticle (N-S1 PNp) that was formed by desolvating N protein into a protein nanoparticle as the core and crosslinking S1 protein onto the core surface against SARS-CoV-2.

**Results:**

Vaccination with N-S1 PNp elicited robust humoral and vigorous cellular immune responses specific to SARS-CoV-2 in mice. Compared to soluble protein groups, the N-S1 PNp induced a higher level of humoral response, as evidenced by the ability of S1-specific antibodies to block hACE2 receptor binding and neutralize pseudovirus. Critically, N-S1 PNp induced Th1-biased, long-lasting, and cross-neutralizing antibodies, which neutralized the variants of SARS-CoV-2 with minimal loss of activity. N-S1 PNp induced strong responses of CD4^+^ and CD8^+^ T cells, mDCs, Tfh cells, and GCs B cells in spleens.

**Conclusions:**

These results demonstrate that N-S1 PNp vaccination is a practical approach for promoting protection, which has the potential to counteract the waning immune responses against SARS-CoV-2 variants and confer broad efficacy against future new variants. This study provides a new idea for the design of next-generation SARS-CoV-2 vaccines based on the B and T cells response coordination.

**Graphical Abstract:**

**Figure:**
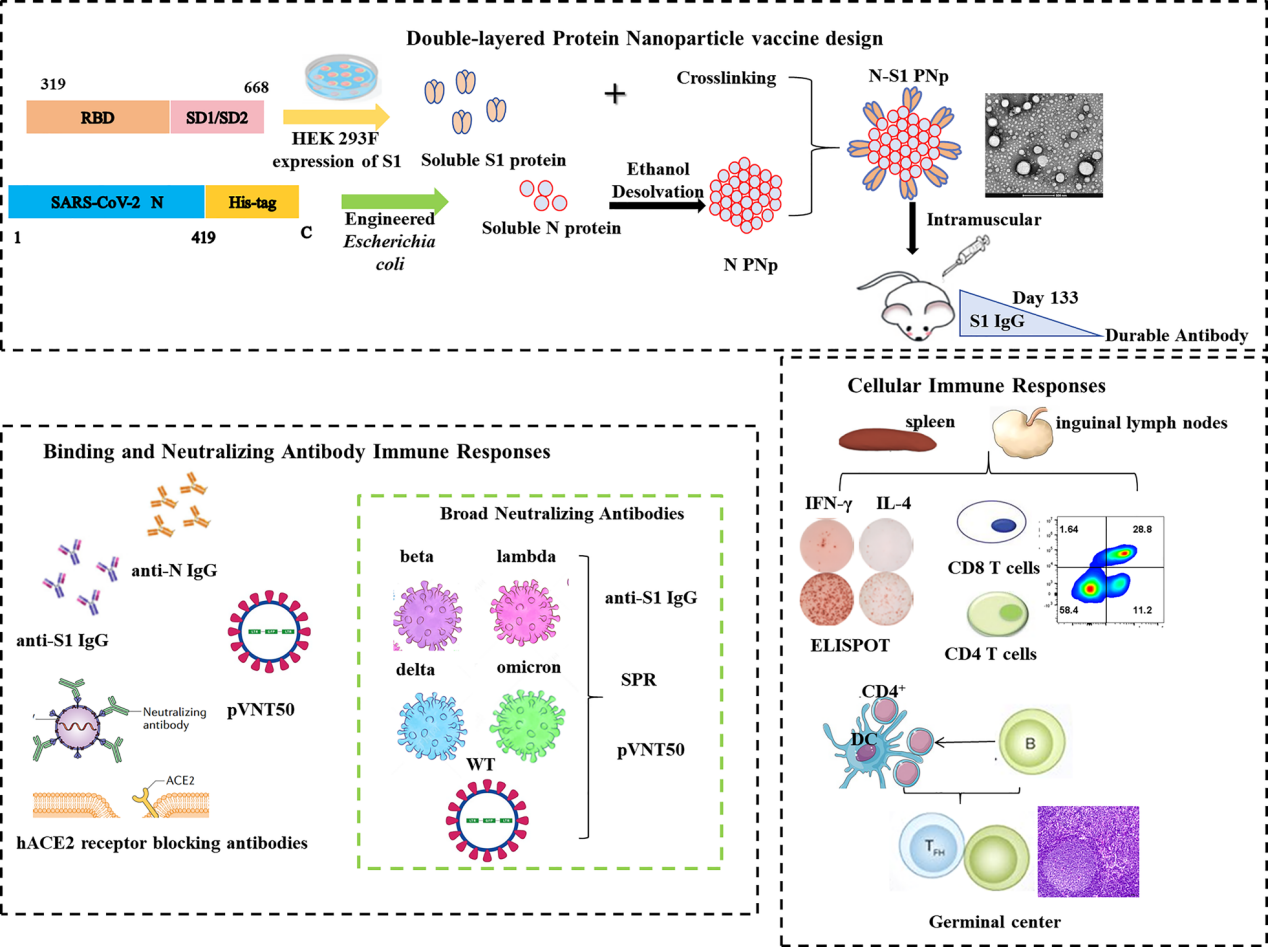
Steps involved in the preparation of double-layered N-S1 protein nanoparticle vaccines and experimental design performed in combating virus infection. After intramuscular immunization of mice, the double-layered N-S1 protein nanovaccine could effectively promote the maturation of antigen-presenting and mature dendritic cells, robust broad-spectrum neutralizing antibody production, cytokines secretion, robust mDC, Tfh cell, and GCs B cell responses induction, T-cell memory formation and durable antibody responses, and unique global transcriptome characteristics, thus achieving a robust cellular immunity and broad antibody responses against SARS-CoV-2 based on the B and T cells response coordination

**Supplementary Information:**

The online version contains supplementary material available at 10.1186/s12951-024-02293-y.

## Introduction

The relentless spreading of SARS-CoV-2 has led to a global pandemic of COVID-19 [1]. Till May 2023, the pandemic has resulted in 760 million infections and 6.92 million deaths globally. Thus, vaccines based on nucleic acid (DNA or RNA), viral vectors, and nanoparticles, play a crucial role in reducing the severity of SARS-CoV-2 and bringing its transmission under control [2].

Various strategies are being employed worldwide to develop COVID-19 vaccine candidates. Currently, the vaccines that have received approval for clinical trials are subunit vaccines (NVX-CoV2373 and ZF2001), mRNA vaccines (mRNA-1273, BNT162b2, and SYS6006), viral vectored vaccines (Ad26.COV2.S, ChAdOx1 nCoV-19, and Convidecia) and inactivated vaccines (CoronaVac and BBIBP-COrV) [2–5]. The subunit vaccine exhibits the highest safety profile among them [5, 6]. However, most developed vaccines primarily target the Spike (S) protein due to its recognition as the principal immune-associated in protecting against SARS-CoV-2 infection [7–9]. In other words, the primary objective of vaccines is to trigger a robust neutralizing antibody for blocking spike-mediated entry. Indeed, the fast growth of the first-generation vaccines provided hope for the ending of the COVID-19 pandemic, but the emergence of variants with greater transmissibility and immune escape ability has presented persistent challenges to vaccine-induced immunity, particularly in the reduced sensitivity of multiple S protein variants to vaccine-induced neutralization [10–12]. For instance, certain vaccines such as ChAdOx1, NVX-CoV2373, and Ad26.COV2.S exhibits varying degrees of reduced cross-reactivity against the beta, delta, and omicron [13]. Similarly, the cross-reactivity of the mRNA vaccine to omicron was reduced by more than 20-fold [14]. Additionally, vaccinated individuals experienced waning immunity over time, meaning that the T-cell response (when present) stimulated by the S-targeted vaccines provides too limited cellular immunity to maintain long-term immune levels [15]. Therefore, it’s imperative to develop more efficient vaccines that can elicit safer, broader, and longer-lasting protection from SARS-CoV-2 and its emerging variants.

Relevant studies have revealed that measurable NAb against Spike was never found in some SARS-CoV-2 infected patients who still eventually recover, suggesting that humoral and cellular immune responses targeting multiple antigens such as S and nucleocapsid (N) antigens in protection against SARS-CoV-2 [7, 16–19]. Nucleoprotein (N) plays a crucial role in the assembly and genome packaging of SARS-CoV-2, which can induce effective T-cell responses during infection [20, 21]. While the spike protein accumulates mutations leading to immune escape, the N protein remains highly conserved among various betacoronaviruses, with an amino acid sequence identity exceeding 90% [22–24]. Therefore, the N protein is considered a promising supplementary target for enhancing vaccine-mediated protective immunity and eliciting broadly-active T cells.

Nanotechnology has been harnessed in developing SARS-CoV-2 vaccines to enhance cellular and humoral immune responses. A vaccine named RBDM, the Prototype RBD-VLP vaccine, based on protein Tag/Catcher technology, had shown high immunogenicity and cross-protection against SARS-CoV-2 emerged variants [25]. Similarly, another independent study investigated CoVLP from Medicago, which was a plant-generated VLP vaccine candidate for COVID-19 and was able to induce better cellular and humoral immune protection [26]. A SARS-CoV-2 spike ferritin nanoparticle vaccine has been found to elicit robust and long-lasting neutralizing antibodies in nonhuman primates [27]. Additionally, a 24-mer RBD-ferritin nanoparticle vaccination resulted in effective cross-neutralizing antibodies against SARS-CoV-2 and batCoVs in the macaque [28]. However, antigen preparation should avoid utilizing large self-assembling structures or nanocarriers, such as ferritin or hepatitis B core protein, which could decrease the risk of off-target immune responses against self-assembly motifs [29, 30].

Herein, we generated a novel double-layered N-S1 protein nanoparticle (N-S1 PNp) vaccine of SARS-CoV-2. The soluble S1 protein and desolvated N PNp were incorporated together to form a self-disassembling layered protein nanoparticle, which composes almost entire antigenic proteins of interest with a tiny amount of crosslinkers for fixation [31–33]. Thus, N-S1 PNp had the highest possible loads of target antigen and was released slowly in the intracellular redox condition. The results demonstrated that the N-S1 PNp exhibited highly immunogenic and elicited robust SARS-CoV-2 antigen-specific B- and T-cell responses in mice, including potent blocking hACE2 receptor antibodies and neutralizing antibodies. To summarize, N-S1 PNp represents a reliable solution for developing a highly effective COVID-19 vaccine against SARS-CoV-2, which also works against emerging SARS-CoV-2 variants.

## Results

### Fabrication and characterization of double-layered N-S1 protein nanoparticle

**Fig. 1:**
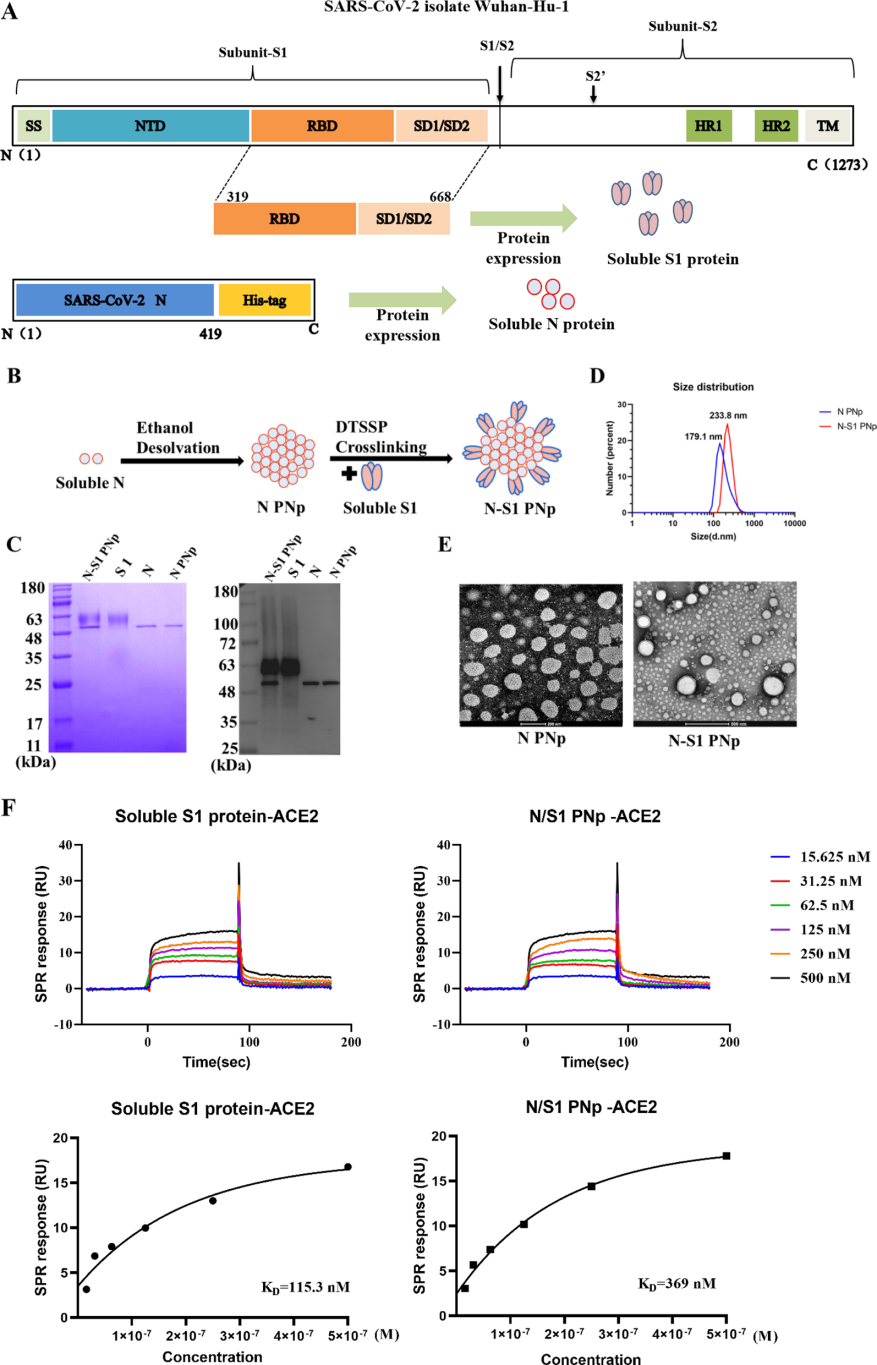
The fabrication and characterization of double-layered N-S1 protein nanoparticle. **A** The schematic diagram of the SARS-CoV-2 isolate Wuhan-Hu-1 spike (top) and nucleocapsid phosphoprotein (bottom), showing signal sequence (SS); N-terminal domain (NTD); receptor binding domain (RBD); subdomains 1 and 2 (SD1/ SD2); furin cleavage (S1/S2); cleavage site (S2′); heptad repeats 1 and 2 (HR1/HR2); transmembrane domain (TM). **B** Schematic diagram of generating double-layered protein nanoparticle. Soluble N protein was self-assembled into PNp by ethanol desolvation. An additional layer of S1 proteins was crosslinked onto the desolvated N PNp particulate core surface as coating via DTSSP crosslinking. **C** Coomassie blue staining and Western blotting analysis of soluble N or S1 protein and protein nanoparticles using anti His-tag monoclonal antibody and S1 monoclonal antibody. Line 1, double-layered N-S1 protein nanoparticle; Line 2, soluble S1 protein; Line 3, soluble N protein; Line 4, N protein nanoparticles. **D** The size spectrum of protein nanoparticles. Red, N-S1 PNp; Blue, N PNp. **E** TEM image of N PNp and N-S1 PNp.Bar scale, 200 nm (left) and 500 nm (right). **F** The affinity of the ACE2 protein to soluble S1 protein or N-S1 PNp was evaluated (top unfitted, bottom affinity). The K_D_ value of soluble S1 protein (left) and N-S1 PNp (right) was determined using SPR

SARS-CoV-2 S1 and N proteins were successfully expressed and purified, observed at approximately 60 kDa and 52 kDa (Fig. 1C). Soluble S1 protein includes an RBD (319–541 AA) derived from the isolate Wuhan-Hu-1 as well as SD1 /SD2 (541–668 AA) sequences (Fig. 1A, top); soluble N protein contains the full-length sequence (1–419 AA) of nucleocapsid phosphoprotein, with a C-terminal six-histidine-tag sequence added (Fig. 1A, bottom). The N protein nanoparticles generated by ethanol desolvation as the core (N PNp). Subsequently, the S1 protein was coated onto the surface of N PNp with 3,3′-dithiobis (sulfosuccinimidylpropionate) (DTSSP) crosslinking to generate N-S1 protein double-layered nanoparticle (N-S1 PNp) (Fig. 1B). The hydrodynamic diameter ranges of N-S1 and N nanoparticles were 220 ± 50 nm and 150 ± 30 nm, as determined by dynamic light scattering (DLS) analysis (Fig. 1D). The Coomassie blue staining and Western blotting analysis proved the S1 and N composition of the double-layered N-S1 PNp (Fig. 1C). The N-S1 PNp can be fully disassembled and reduced to monomers. The overall percentage of S1 in its layered protein nanoparticles was 69% ± 1.9% through Image J intensity analysis of Coomassie Blue-stained gel protein bands (Fig. 1C). Transmission electron microscopy revealed the double-layered protein N-S1 nanoparticle to be approximately spherical, with mostly irregular morphology (Fig. 1E). The affinity of soluble S1 protein and N-S1 PNp to ACE2 protein(the cellular receptor for SARS-CoV-2) was tested via surface plasmon resonance assay (SPR). As expected, the binding curve was fitted by the "Steady State Affinity" analysis (Fig. 1F). The ACE2 protein exhibited high affinity binding to the soluble S1 protein and N-S1 PNp, namely 115.3 nM and 369 nM. The results demonstrated that the S1 protein coated onto the surface of N PNp in our design would not interfere with the solution exposure of its receptor binding motif.

### Double-layered N-S1 PNp effectively promoted BMDCs uptake and maturation in vitro

**Fig. 2:**
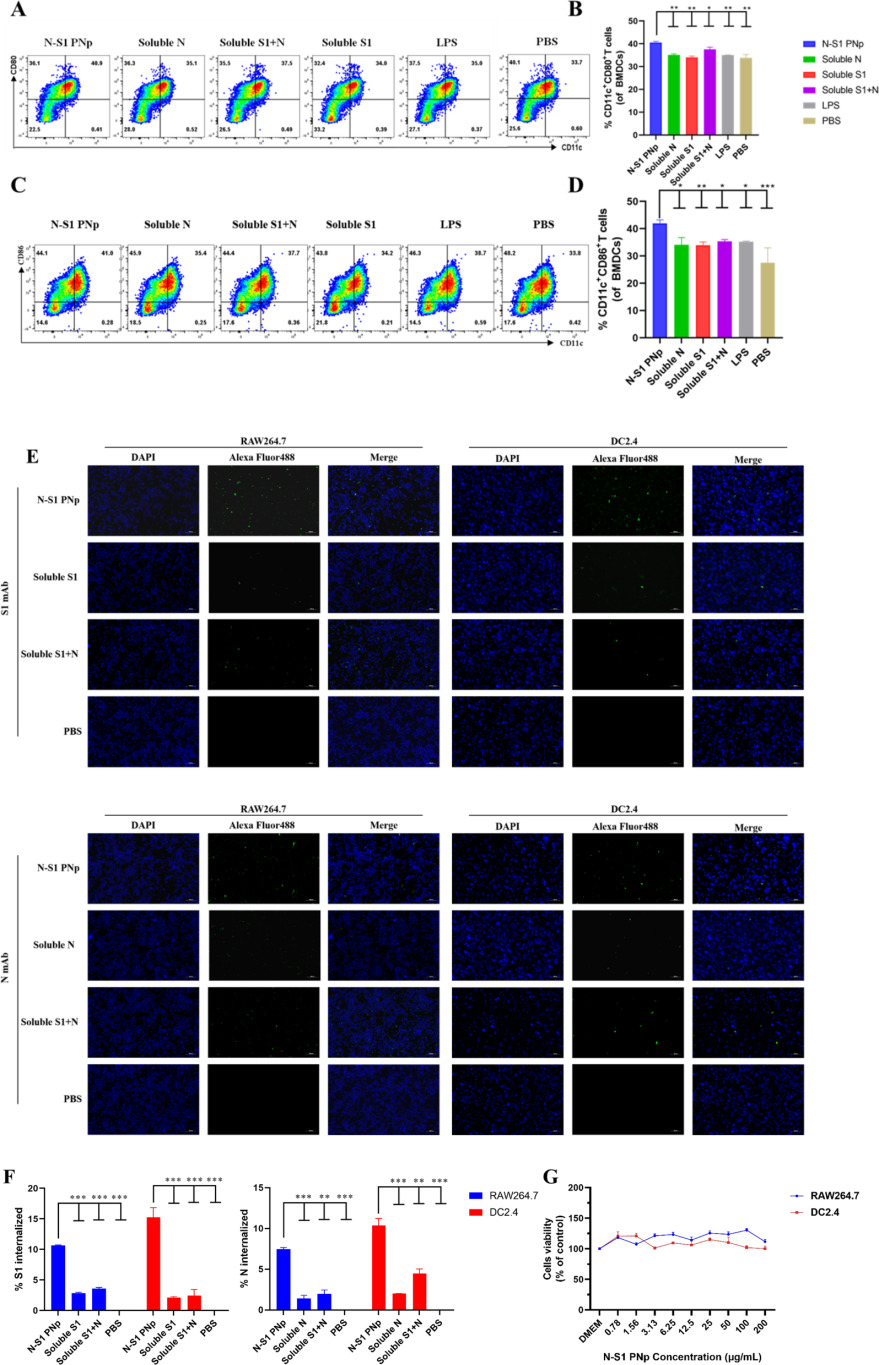
Double-layered N-S1 PNp on the maturation, uptake, and cytotoxicity of APCs were determined in vitro. **A–D** The expression of CD11c^+^, CD80^+^, and CD86^+^ surface markers on BMDCs. **A**, **C** Representative pseudocolor dots in different groups. **B**, **D** The percentages of CD11c^+^, CD80^+^, and CD11c^+^, CD86^+^ were measured using FCM. **E**, **F** In vitro APCs uptake assay. **E** Representative Immunofluorescent staining images of RAW264.7 (left) and DC2.4 (right) cells in different groups. From left to right, the panel showcases DAPI (blue) on the left, Alexa Fluor 488 labeled S1 (top) and N (bottom) (green), followed by a merged image of the two. The scale represents 100 μm. **F** The percentage statistics of internalized S1 (left) and N (right). Bars are presented as mean ± SD of triplicate technical replicates. Statistical significance was conducted by one-way ANOVA analysis followed by multiple comparisons post hoc tests (***p-value < 0.001, **p-value < 0.01, *p-value < 0.05, ^ns^p-value > 0.05). **G** The cytotoxicity of N-S1 PNp to APCs in vitro

To investigate the impact of double-layered N-S1 PNp on DCs maturation, detected the expression of co-stimulatory molecules associated with DCs maturation in vitro using BMDCs. Compared to other treatments, double-layered N-S1 PNp—treated BMDCs exhibited the higher expression of CD11c^+^ CD80^+^, and CD11c^+^ CD86^+^ DCs, indicating its potent ability to promote BMDCs maturation (Fig. 2A–D).

Quick uptake by antigen-presenting cells (APCs), including macrophages and dendritic cells, is pivotal in inducing a robust immune response. RAW264.7 and DC2.4 cell lines are commonly utilized models to assess the level of antigen uptake by APCs. Compared with PBS, green fluorescence was distributed in the cytoplasm representing S1 or N protein when evaluating the cellular uptake level of double-layered N-S1 PNp in RAW264.7 and DC2.4 cells (Fig. 2E). The results showed that the double-layered N-S1 PNp group had higher fluorescence intensity compared to any soluble antigen, indicating N-S1 PNp was more effectively internalized into the APCs in vitro (Fig. 2F). The safety of double-layered N-S1 PNp was further evaluated in vitro using the CCK-8 assay. The results showed that the cell viability was not less than 100% even at the double-layered N-S1 PNp concentration up to 200 μg/mL, which substantiated that double-layered N-S1 PNp had no cytotoxicity effect on RAW264.7 cells and DC2.4 cells (Fig. 2G).

### Double-layered N-S1 PNp immunization elicited robust binding and neutralizing antibody immune responses in mice

**Fig. 3:**
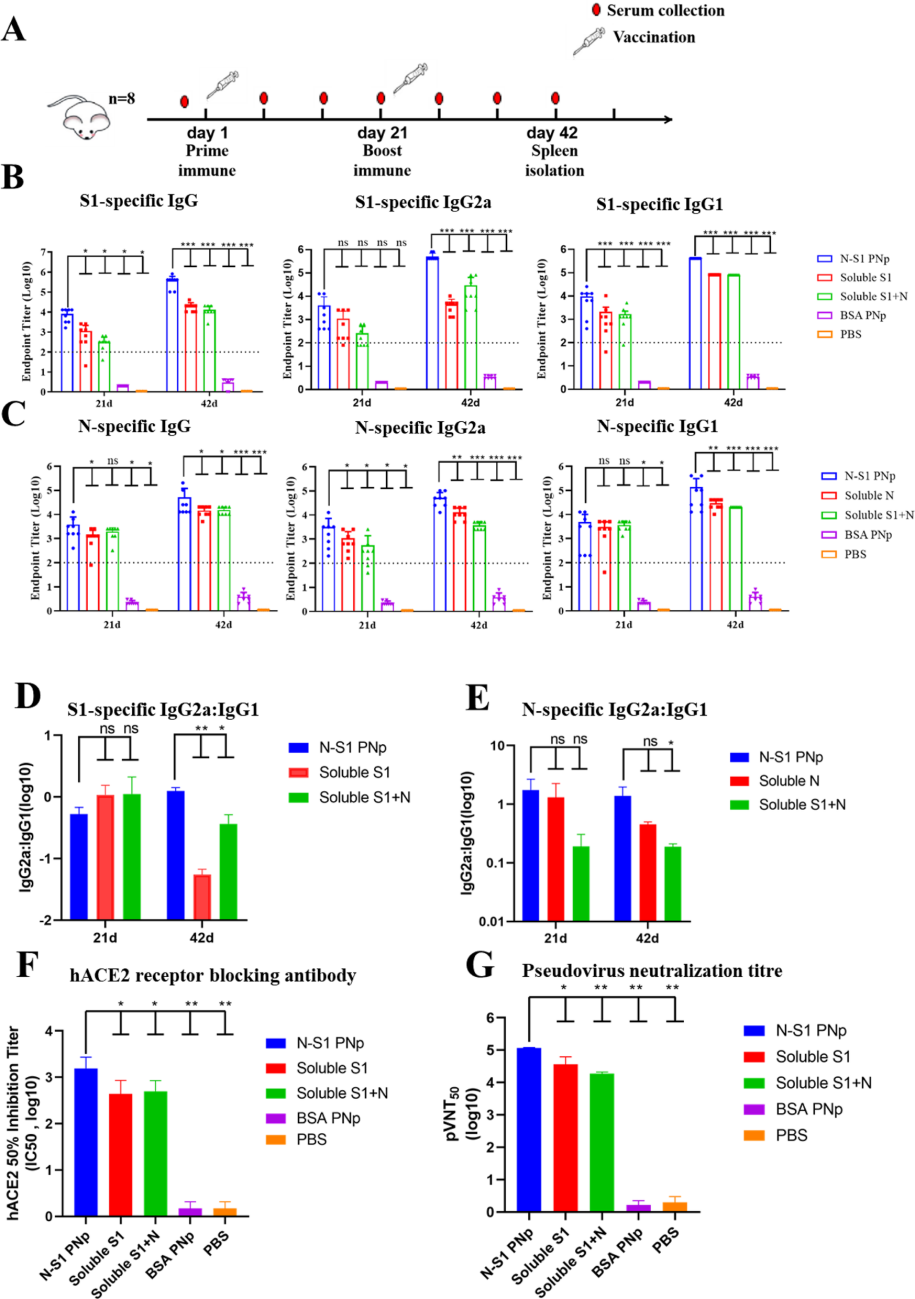
Humoral immune responses were assessed in immunized mice. **A** The experimental timeline and sample collection protocol for vaccinating BALB/c mice. **B**, **C** Serum specimens were determined for SARS-CoV-2 S1 and N protein-specific IgG, IgG2a, and IgG1 endpoint titers by ELISA. **D**, **E** Bar chart of logarithm values of the ratio IgG2a: IgG1 of S1 and N protein. Data are reported as mean ± SD (n = 8). Comparisons were performed by one-way ANOVA with the Bonferroni correction in SPSS 21.0. ***p-value < 0.001, **p-value < 0.01, *p-value < 0.05, ^ns^p-value > 0.05. Data were combined from three parallel replicates per sample. Colored symbols indicated individual animals’ values. **F** Antibodies that block the hACE2 receptor in pooled serum (n = 8/group) collected at 21st days post-boost vaccination. **G** SARS-CoV-2 spike pseudovirus 50% serum neutralizing antibody titers (pVNT50) in pooled serum (n = 8/group) collected from (**F**). Bars indicate the mean ± SD of three parallel replicate assays per sample

To assess the immunogenicity of double-layered N-S1 PNp, groups of mice (n = 8/group) were intramuscularly immunized twice with a 3-week interval. Vaccines were formulated with aluminum hydroxide and CPG 1018 adjuvants (Fig. 3A). The Humoral immune response was evaluated by analyzing sera collected from mice at different time points. Immune serum antibody levels showed that strong seroconversion against S1 and N was elicited 3 weeks after the priming/boosting immunizations in the double-layered N-S1 nanoparticles group (Fig. 3B–E). Compared with the other immunization groups, mice immunized with the double-layered N-S1 nanoparticles immunizations had significantly increased S1 and N protein-specific IgG levels, as well as higher titers of IgG1 and IgG2a subtypes (geometric mean titer of IgG, IgG GMTs = 688 000 and 51 200, respectively) (Fig. 3B, C). Importantly, the levels of IgG1 and IgG2a of the immune response were assayed using ELISA, which were associated with Th2 and Th1 immunity [34–36]. The subtyping analysis divulged that immunization with the double-layered N-S1 nanoparticles induced IgG2a-biased antibody responses, as evidenced by the ratio of IgG2a to IgG1 (Fig. 3D, E), which has suggested a Th1-bias response.

Furthermore, the human ACE2 (hACE2) was identified as the primary functional receptor for SARS-CoV-2 viral entry. Antisera from mice immunized with the double-layered N-S1 nanoparticles at day 21st post-boost immunization elicited high titer antibodies that effectively inhibited the binding of S1-protein to hACE2 receptor (The average IC50 = 1550) (Fig. 3F). The serum obtained from mice immunized with the double-layered N-S1 nanoparticle could strongly block hACE2 receptor binding to S1-protein on the surface of SARS-CoV-2. Moreover, the blocking effect was significantly higher than that of soluble protein groups.

Additionally, the serum from vaccinated mice was assessed for its ability to neutralize using a SARS-CoV-2 spike pseudovirus assay. Two doses of N-S1 protein double-layered nanoparticle, soluble S1 protein, a formulation comprising a protein mixture of soluble S1 protein and soluble N protein elicited 50% pseudovirus neutralization antibody geometric mean titers (pVNT50 GMTs) of 116,305, 27,578 and 18,896, respectively, 21 days after boost immunization (Fig. 3G). The group of N-S1 PNp vaccinated animals exceeded that of the other two groups by a factor of 3.2 and 6.15 (Fig. 3G).

### Double-layered N-S1 PNp vaccinated mice enhanced systemic cellular immune responses

**Fig. 4:**
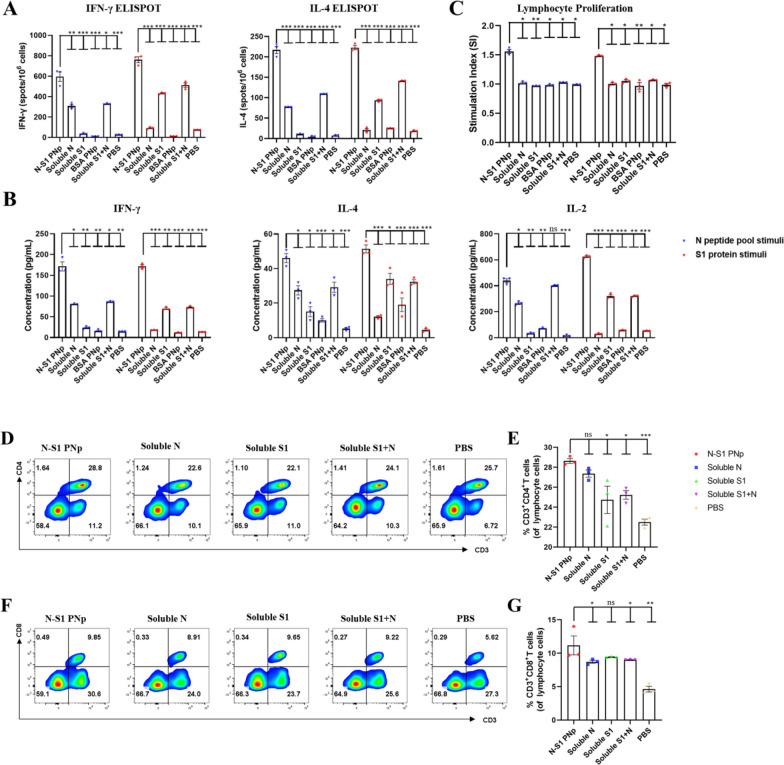
Evaluation of splenic cellular immune responses in immunized mice. **A** Enumeration of Interferon-gamma (IFN-γ) and Interleukin-4 (IL-4) secreting splenocytes clones re-stimulated with N peptide pool or S1 protein were determined by ELISpot assay.** B** The levels of IFN-γ, IL-4, and IL-2 secreted in the splenocytes culture medium from immunized mice were detected by cytokine ELISA after stimulation for 2.5 days. **C** The SI of lymphocyte proliferation assay. **D–G** Splenocytes were analyzed in FCM. Percentage of activated CD4^+^ (**E**) and CD8^+^ (**G**) T cells. **D**, **F** Representative pseudocolor plots (smooth on). Bars are presented as mean ± SEM of triplicate assays, where n = 3 mice in each group. Individual values are indicated by colored symbols. Statistical significance was assessed by one-way ANOVA analysis followed by multiple comparisons post hoc tests in A-B, and corresponding P values shown in bar charts. ***p-value < 0.001, **p-value < 0.01, *p-value < 0.05, ^ns^p-value > 0.05

Cellular responses contribute to the killing of pathogens and the generation of effective immunity [37, 38]. ELISpot analysis of IFN-γ and IL-4 on lymphocytes from immunized animals spleens were stimulated with synthetic N peptides pool or S1 protein. Splenocytes from mice immunized with the N-S1 protein double-layered nanoparticle group augmented IFN-γ and IL-4 secretion, which showed the highest number of IFN-γ and IL-4 secreting cells after the boost immunization in the study (Fig. 4A). We further measured the cytokine levels in the splenocyte culture medium after stimulation (Fig. 4B). The N-S1 protein double-layered nanoparticle-immunized group showed the highest production of recall cytokines IFN-γ, IL-4, and IL-2 in the medium. These results imply that the N-S1 protein double-layered nanoparticle can regulate Th1 (the TH1 cytokines IL-2 or IFN-γ) and Th2 (one of the TH2 cytokines IL-4) immunity simultaneously without the excessive release of inflammatory factors. It is generally known that the proliferation of spleen lymphocytes reflects the host immune status [39]. We performed the lymphocyte proliferation assay under the N peptides pool or S1 protein re-stimulated for 60 h. Our data suggest that the N-S1 protein double-layered nanoparticle-immunized can promote the proliferation of lymphocytes, and the stimulation index (SI) was significantly greater than the other groups (Fig. 4C).

CD4^+^ T cells and CD8^+^ T cells are key immune cells in SARS-COV-2 specific adaptive immune response. The CD4^+^ T cells play a pivotal role in promoting B cell differentiation and sustaining the response of CD8 cytotoxic T cells. Hence, the T cell response in the spleen elicited by double-layered N-S1 PNp was further evaluated using flow cytometry (FCM). The content of CD3^+^ CD4^+^ double-positive T cells in mice immunized with the double-layered N-S1 protein nanoparticle three weeks after boost vaccine doses significantly increased (Fig. 4D, E). While CD3^+^ CD8^+^ double-positive T cells showed the highest expression increase in the layered N-S1 nanoparticle vaccinated group compared to other groups (Fig. 4F, G). These results suggest that the double-layered N-S1 protein nanoparticle immunization could significantly enhance the proliferation and activation of CD4^+^ and CD8^+^ T cell responses, which induced even stronger cellular immunity.

### Double-layered N-S1 PNp vaccines efficiently induce robust mDC, Tfh cell, and GCs B cell responses

**Fig. 5:**
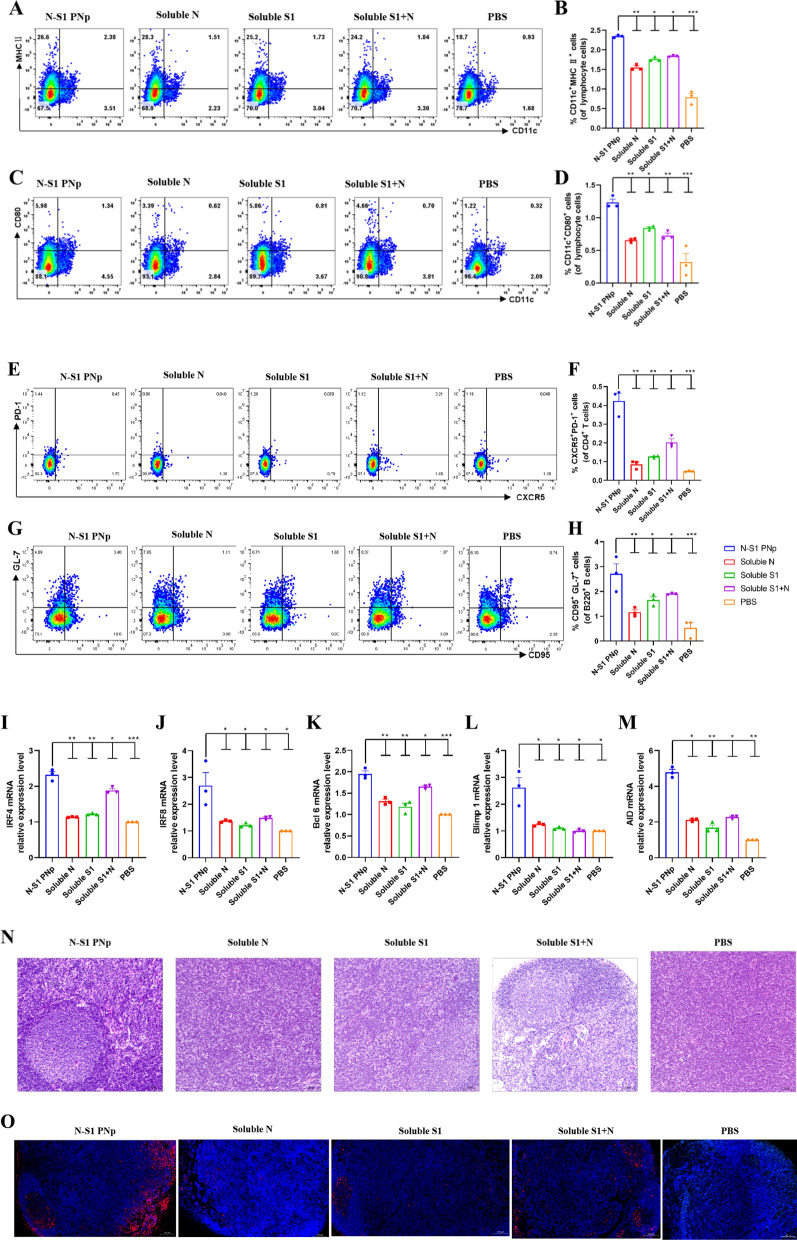
B and T cell responses occurred 21 days after boost immunization. **A–D** Vaccines-induced DCs maturation in vivo. **A**, **C** Representative pseudocolor dots in different groups.** B** The percentages of CD11c^+^, MHCII^+^, and (**D**), CD11c^+^, CD80^+^ were measured using FCM. **E**, **F** Splenic Tfh cells(CD4^+^ CXCR5^+^ PD-1^+^) representative FCM pseudocolor dots (**E**) and statistic percentages in the CD4 T population (**F**). **G**, **H** Splenic germinal center (GCs) B cells (GL7^+^ CD95^+^ B220^+^) representative FCM pseudocolor dots (**G**) and statistic percentages in the B220 B population (**H**). **I**, **J** Relative expression levels of IRF4 (**I**) and IRF8 (**J**) related to DC regulation. **K**, **L** Relative expression levels of Bc16 (**K**) and Blimp 1 (**L**) related to Tfh transcriptional regulators. **M** The relative expression level of AID associated with GCs B cells activation. Bars are presented as mean ± SEM of triplicate assays, where n = 3 mice in each group. Individual values are indicated by colored symbols. Statistical significance was conducted using one-way ANOVA analysis followed by multiple comparisons post hoc tests, and P values shown in bar charts. ***p-value < 0.001, **p-value < 0.01, *p-value < 0.05, ^ns^p-value > 0.05. **N**, **O** The formation of GCs was observed by H&E staining (**N**, Bar represents 50 μm) and B220 immunofluorescence staining (**O**, Bar represents 100 μm) in inguinal lymph nodes harvested from immunized mice

Double-layered N-S1 PNp was able to generate high levels of specific antibodies (Fig. 3), and sufficient CD4^+^ follicular help T cells (Tfh) play a fundamental role in eliciting and maintaining high-affinity antibody responses. DCs maturation has a crucial impact on inducing the generation of the humoral immune response, driving T cells to differentiate into Tfh cells. Thus, the DCs maturation was further investigated in vivo by FCM and real-time qPCR (RT-qPCR). Splenic dendritic cells were isolated from different immunized mice at 3 weeks after boost immunization. FCM showed that compared with other groups, N-S1 PNp-immunized induced higher expression levels of MHCII and CD80, which were the DCs maturation stimulatory marker (Fig. 5A–D). Besides, RT-qPCR indicated significant upregulation of IR4 and IRF8 relative expression levels in N-S1 PNp-immunized mice compared to other groups, which were the crucial regulators of DC cell differentiation (Fig. 5I, J). Double-layered N-S1 PNp immunity could significantly promote DCs maturation of the spleen in vivo.

Subsequently, we evaluated the CD4^+^ Tfh cells frequency in spleens. Spleen lymphocytes were isolated after 21 days post-boost vaccination, and Tfh cell responses were detected using FCM and RT-qPCR. Animals immunization with double-layered N-S1 PNp significantly increased Tfh cells frequency (CD4^+^ CXCR5^+^ PD-1^+^) in the spleen (Fig. 5E, F). Moreover, Blimp 1 and Bc16 are transcription regulatory factors related to Tfh, which play a decisive role in the normal differentiation and development of Tfh. Analysis of Bc16 and Blimp 1 relative expression levels, using RT-qPCR, yielded similar findings (Fig. 5K–L).

Due to the assistance provided by Tfh cells to B cells in GCs, which play a crucial role in regulating high-quality antibody responses, we further assessed B cell maturation and GCs formation. Animals immunized with double-layered N-S1 PNp presented a significantly higher percentage of GCs B cells (B220^+^ GL7^+^ CD95^+^) in spleens compared to other groups(Fig. 5G, H). The relative expression levels of AID induced by activated mature B cells were also measured using RT-qPCR, which yielded similar findings (Fig. 5M). In addition, the formation of GCs was observed in paraffin sections of inguinal lymph nodes using H&E staining and immunofluorescence labeling. The analysis revealed that in the double-layered N-S1 PNp immunized mice, lymphocyte proliferation substantially accumulated in basophilic clumped areas was observed by H&E staining to form a distinct germinal center structure (Fig. 5N). Moreover, the specific molecule B220 of B cells was labeled with CY3 fluorescence and expressed in a circular form (Fig. 5O). Thus, double-layered N-S1 PNp enhances GCs B cell maturation.

### Double-layered N-S1 PNp promoted T-cell memory formation and durable antibody responses

**Fig. 6:**
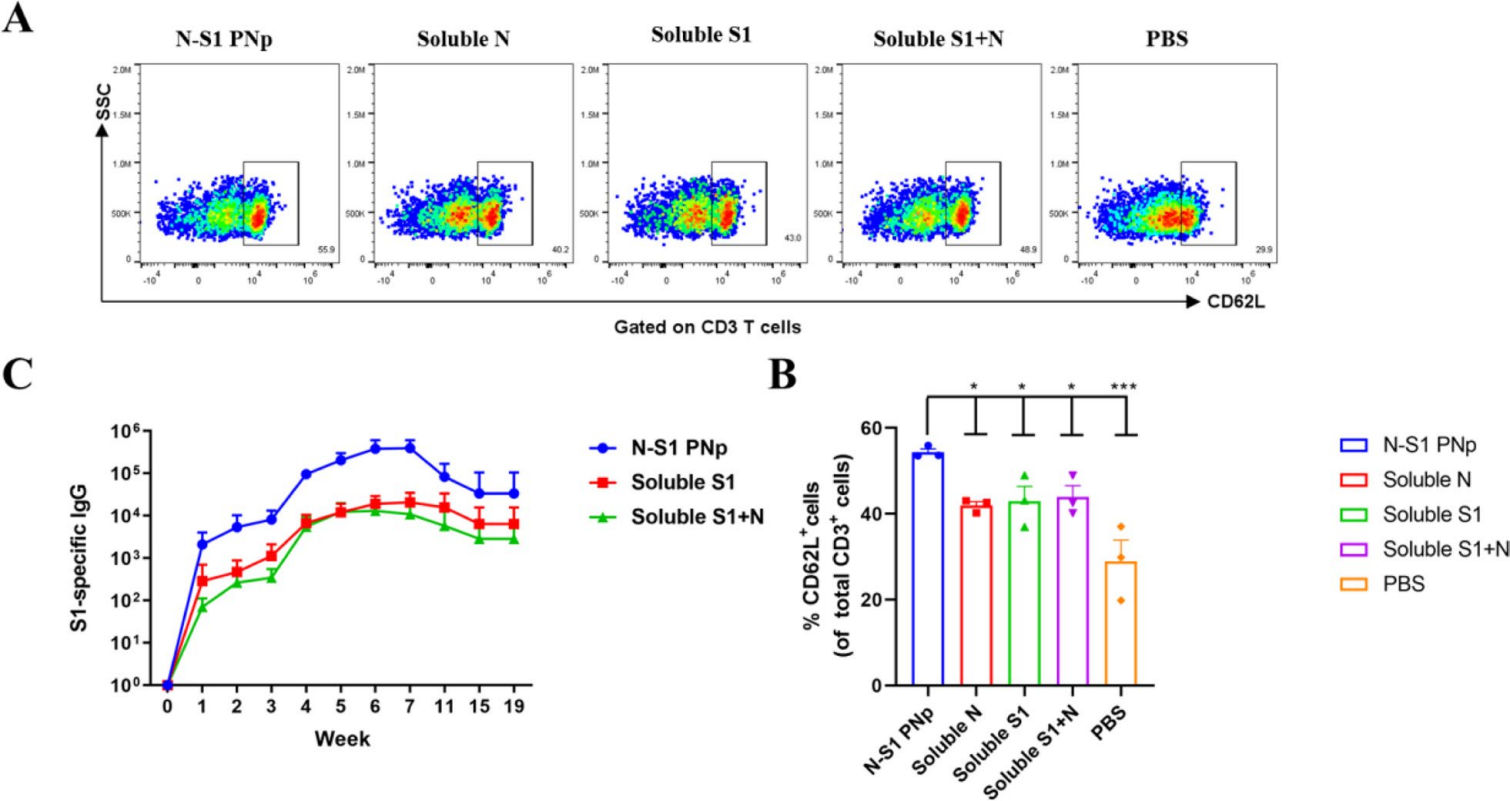
Long-term immunity induced by double-layered N-S1 PNp vaccination. **A**, **B** Robust the central memory of CD3 T cells in spleens. **A** Representative pseudocolor dots in different groups. **B** The percentages of CD62L^+^ were measured using FCM. Bars are presented as mean ± SEM of triplicate assays, where n = 3 mice in each group. Colored symbols indicate individual values, while statistical significance was conducted through one-way ANOVA analysis followed by multiple comparisons post hoc tests. The corresponding P values display in bar charts. ***p-value < 0.001, **p-value < 0.01, *p-value < 0.05, ^ns^p-value > 0.05. **C** Serum S1-specific antibody response was continuously detected weekly by ELISA

Vaccination with double-layered N-S1 PNp has shown enhancement to DCs maturation, as well as Tfh cells and GCs B cells development. Meanwhile, durable antibody responses are associated with mediating of GCs reactions. And the Tfh cell response induced by vaccines may boost long-term immunity, thereby thwarting SARS-CoV-2 infection [40, 41]. Boosting local and systemic memory T-cell responses is an effective strategy for achieving long-term immunity, a necessary feature of an ideal vaccine. Therefore, we assessed central memory T cells activation in spleens using FCM from immunized mice on day 49th since the first vaccination. Subsets of CD3^+^ central memory T cells (CD3^+^ CD62L^+^) significantly elevated in mice vaccinated with the double-layered N-S1 PNp vaccine (Fig. 6A, B). Moreover, we evaluated the durability of the immunity elicited by the double-layered N-S1 PNp. The follow-up study on antibody titer was conducted, and the sera of immunized mice were collected weekly. Then antibody endpoints against the S1 protein were measured by indirect ELISA. N-S1 PNp-induced antibodies specific to the S1 protein exhibited durability in mice for up to 4 months after the final immunization, remaining at high antibody levels (Fig. 6C).

### Double-layered N-S1 PNp induced robust and broad neutralizing antibodies against SARS-CoV-2 prototype (WT) and variants

**Fig. 7:**
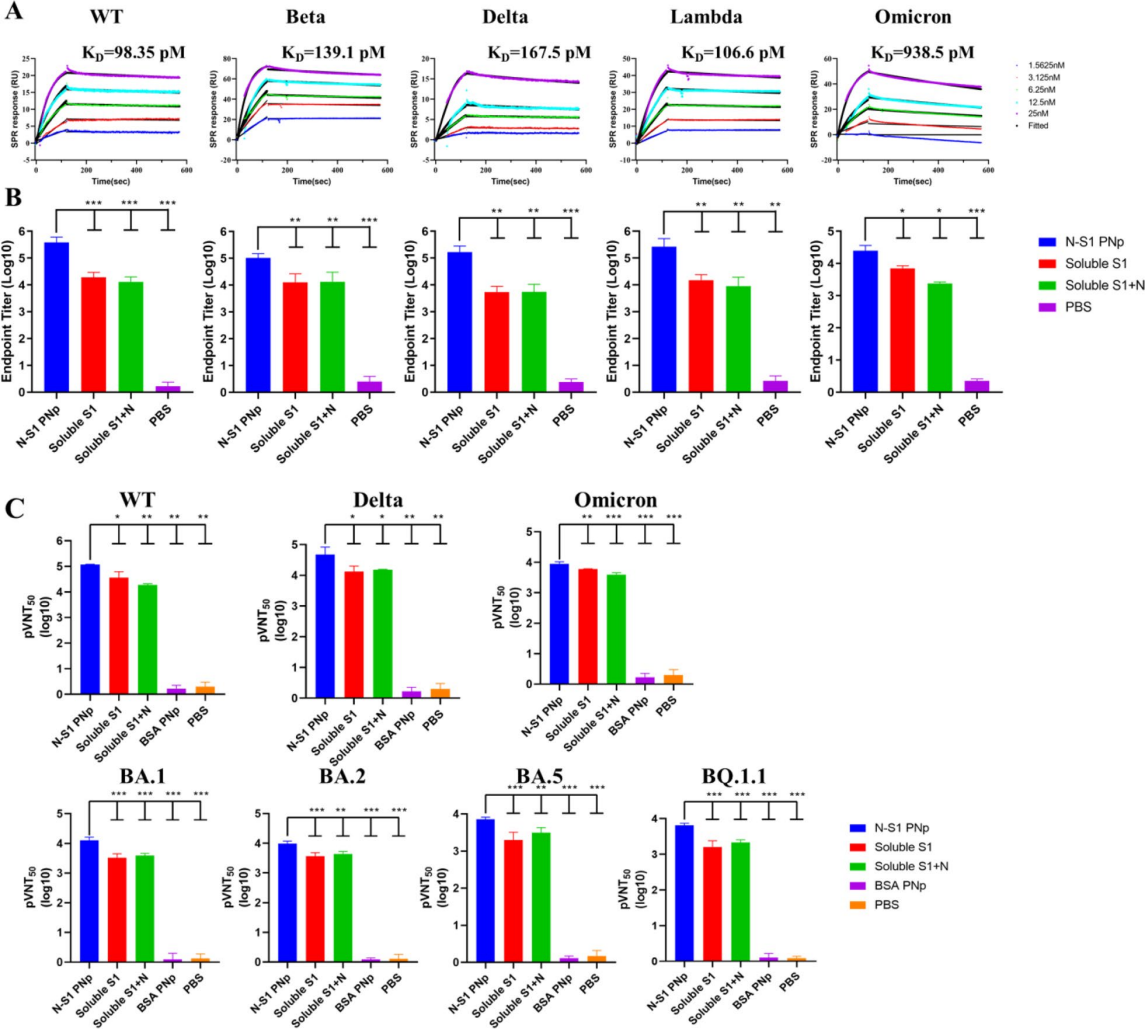
Neutralizing antibody responses were elicited against SARS-CoV-2 isolate Wuhan-Hu-1 and variants, including B.1.351, B.1.617.2, C.37, B.1.1.529, BA.1, BA.2, BA.5, and BQ.1.1 by double-layered N-S1 PNp vaccination in mice.** A** Representative SPR sensorgram showing binding kinetics of SARS-CoV-2 WT and its four variants S1 protein to immobilized mouse IgG from serum 21 days after boost immunization with N-S1 PNp (n = 8). The actual binding (color) and the optimal fit of data to a 1:1 binding model (black) are displayed, along with corresponding K_D_ values. **B** The endpoint titers of serum binding to SARS-CoV-2 WT and its four variants of S1 protein were evaluated using indirect ELISA. **C** The pVNT50 of SARS-CoV-2 spike pseudovirus, including WT, B.1.617.2, B.1.1.529, BA.1, BA.2, BA.5, and BQ.1.1 were measured 3 weeks after the final N-S1 PNp vaccination for serum neutralizing antibody titers. Bars represent the mean ± SD of triplicate assays. Statistical significance was conducted using one-way ANOVA analysis followed by multiple comparisons post hoc tests, with corresponding P values displayed in bar charts. ***p-value < 0.001, **p-value < 0.01, *p-value < 0.05, ^ns^p-value > 0.05

The spike glycoprotein of SARS-CoV-2 variants harbors diverse amino acid substitutions, deletions, and insertions, which has raised grave immune escape. Therefore, the SARS-CoV-2 vaccine under development must confer robust protection against WT and its variants. Accordingly, we further assessed the serum-neutralizing antibody responses and binding affinity elicited by the N-S1 PNp vaccine against SARS-CoV-2 WT and its eight variants, including B.1.351 (Beta), B.1.617.2 (Delta), C.37 (Lambda), B.1.1.529 (Omicron), BA.1 (Omicron sub-variant lineage), BA.2 (Omicron sub-variant lineage), BA.5 (Omicron sub-variant lineage), BQ.1.1 (Omicron sub-variant lineage). Serum binding affinity assessment by surface plasmon resonance (SPR) revealed that N-S1 PNp vaccine-elicited IgG could bind strongly to the S1 of WT and four variants with an affinity around 1 nM or lower (Fig. 7A). However, compared with WT, the binding affinity to beta, delta, lambda, and omicron showed a significant decrease, K_D_ values were 98.35 pM, 139.1 pM, 167.5 pM, 106.6 pM, and 938.5 pM, respectively (Fig. 7A). N-S1 PNp induced high levels of potent and broad-spectrum neutralizing antibodies against SARS-CoV-2 WT and its eight variants (Fig. 7B, C). But the neutralization abilities against all variants in the panel were significantly lower than WT (Fig. 7B, C). The reduction of pVNT50 was more pronounced in Delta, Omicron, and Omicron sub-variant lineages (BA.1, BA.2, BA.5, and BQ.1.1), particularly the latter when compared to that of WT. Notably, the serum antibodies of the N-S1 PNp-vaccinated group still exhibited enhanced neutralization activity against Delta, Omicron, and Omicron sub-variant lineages variants in comparison with soluble protein groups (Fig. 7C).

### Double-layered N-S1 PNp immunization induces unique global transcriptome characteristics

**Fig. 8:**
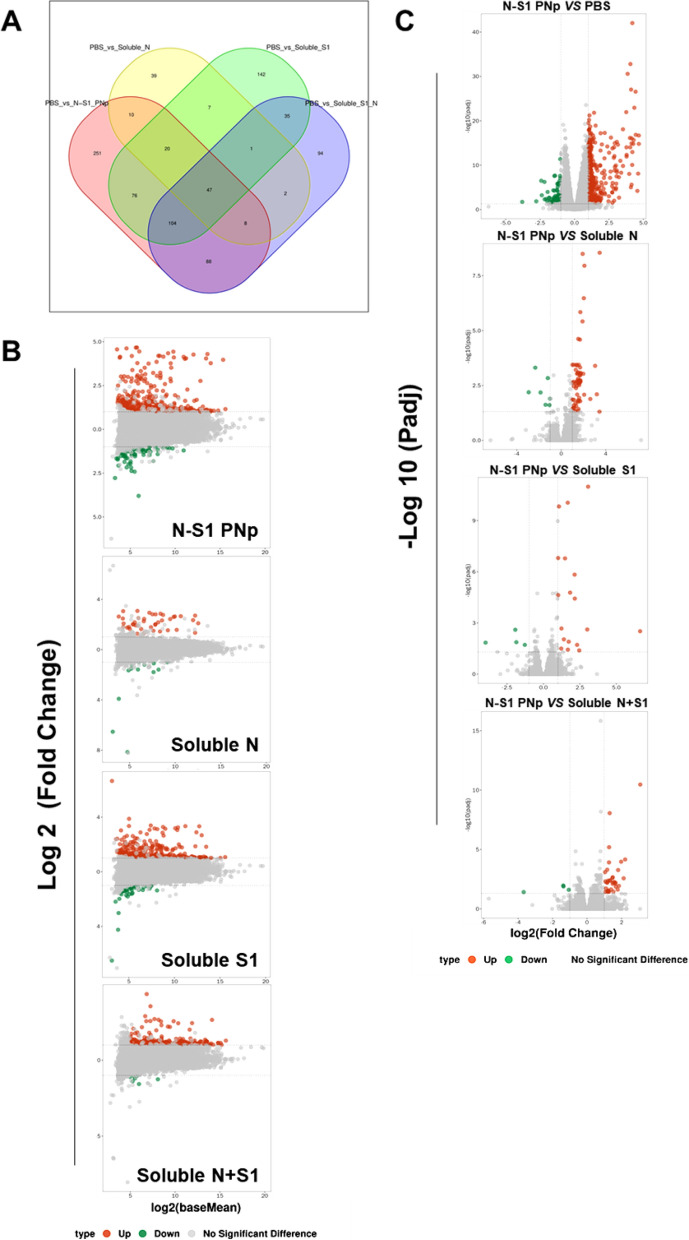
N-S1 PNp immunization altered transcriptional characteristics. **A** Venn diagrams of differentially expressed genes (DEGs) compared to PBS, numbers indicate unique and common DEGs in four groups. **B** MA maps displaying DEGs of PBS versus N-S1 PNp, Soluble N, Soluble S1, and Soluble S1 + N. **C** Volcano maps showing differential gene expression of N-S1 PNp versus PBS, Soluble N, Soluble S1, and Soluble S1 + N. The splash represents various genes, with significant (p < 0.05) up-regulated and down-regulated DEGs represented by red or green dots, respectively, while gray dots represent genes with no significant change

RNA-seq was employed to further study the mechanism underlying the robust immune response induced by double-layered N-S1 PNp. Spleen samples were obtained from mice 21 days post-booster immunization for transcriptomic sequencing, constructed fifteen cDNA libraries, generating 680 million raw reads. After fastp filtering, 679 million high-quality clean reads (99.79% of the raw reads) were obtained and mapped to the reference genome of Mus musculus. There was no significant deviation in the coverage of the sequencing data, indicating that the obtained sequences evenly covered all positions of the reference genome (Additional file 1: Fig. S1B). Gene expression profile analysis of the control group (PBS) versus other groups showed that PBS versus N-S1 PNp markedly exhibit the highest number of significantly differentially expressed genes (DEGs), including up-regulated and down-regulated (Fig. 8A, Additional file 1: Fig. S1C). And more significantly upregulated genes than down-regulated in all groups (Fig. 8A, B, Additional file 1: Fig. S1C). Overall, the results indicate that N-S1 PNp has an impact on transcriptome characteristics. To analyze DEGs between N-S1 PNp and other groups, online software DESeq2 was utilized for gene differential expression analysis, with p-adj < 0.05 and | log2FoldChange |> 1. As shown in the volcano map, in the N-S1 PNp group, 484 were up-regulated and 120 were down-regulated genes compared to the control group. In comparison to the soluble N group, there were 134 up-regulated and 29 down-regulated genes; while in comparison to the soluble S1 group, there were 85 up-regulated and 22 down-regulated genes. Additionally, when compared with the soluble N + S1 group, there were 112 up-regulated and 33 down-regulated genes (Fig. 8C, Additional file 1: Fig. S1D).

**Fig. 9:**
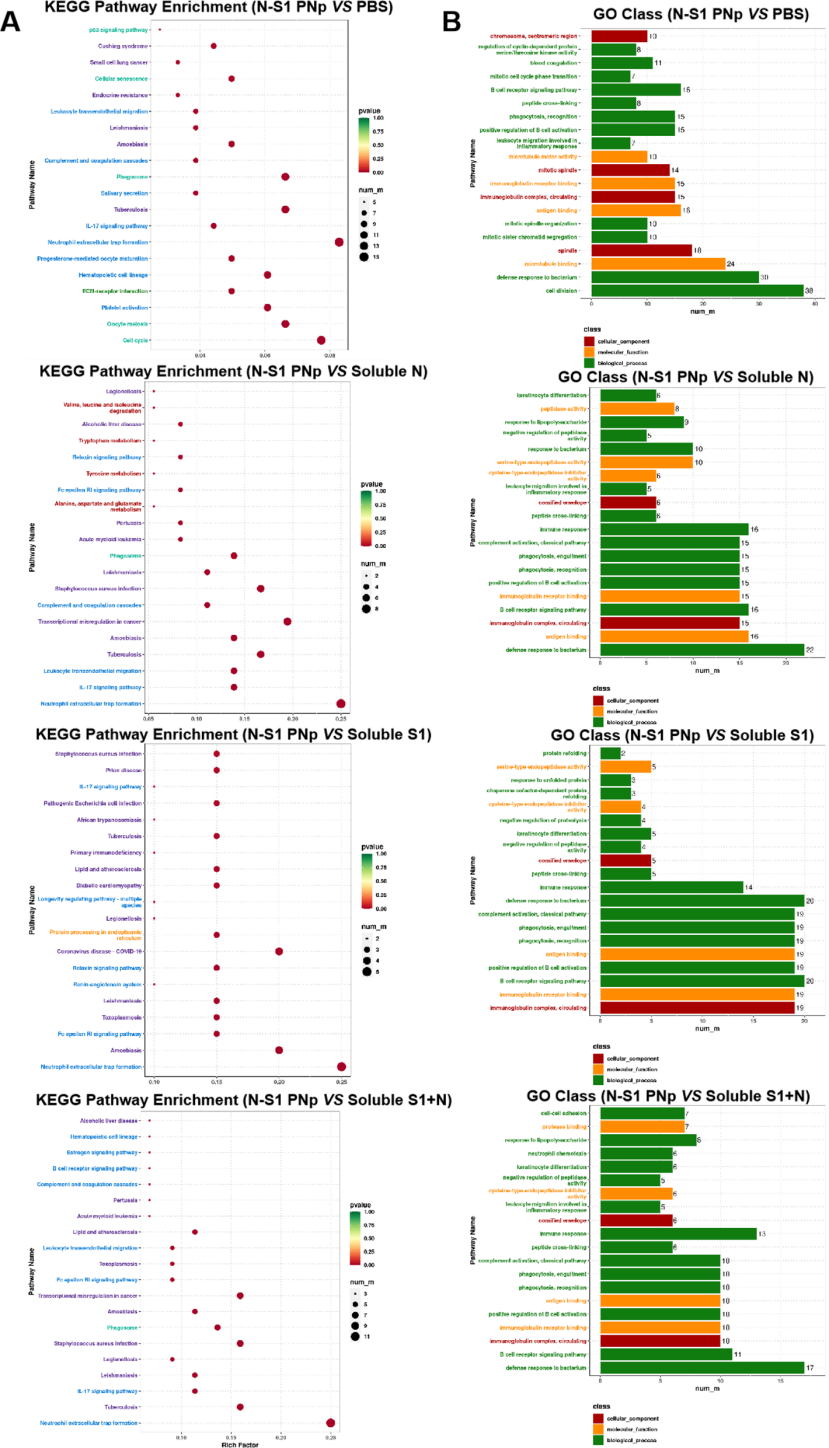
GO and KEGG enrichment analysis of DEGs. The KEGG enrichment bubble plot **A** and GO enrichment analysis **B** of N-S1 PNp versus PBS, soluble N, soluble S1, and soluble S1 + N, ranking the top twenty annotations were listed based on correlation with each functional annotation. The vertical axis is the pathway name, the size of the circle or the horizontal axis represents the number of DEGs, the color represents the richness factor, and the color is the rich factor, representing P.adjust

Further, GO and KEGG enrichment analysis determined the putative functions of DEGs. The top 20 pathways with the most enriched were presented and identified based on rich factor, P.adjust, and the number of genes. P.adjust (value range 0–1) is a corrected P-value that accounts for multiple hypothesis testing; the closer it is to zero, the more significant the enrichment. The GO enrichment results revealed significant enrichment of terms related to immune activation, such as B cell receptor signaling pathway, leukocyte migration involved in inflammatory response, and positive regulation of B cell activation. Most of the top 20 enriched terms were associated with the immune system (Fig. 9B, Additional file 1: Fig. S2B). Similar findings were observed in the KEGG enrichment bubble plot, which primarily involved Neutrophil extracellular trap formation, IL-17 signaling pathway, Phagosome, and Fc epsilon RI signaling pathway (Fig. 9A, Additional file 1: Fig. S2A). IL-17 is predominantly secreted by activated CD4^+^ T cells that produce various cytokines, such as IL-6 and GM-CSF, to recruit neutrophils for promoting inflammatory response. It serves as the main effector of Th17 cells, which exhibit the phenotype of activated memory cells expressing CD69 and CCR7 and are related to central memory T cells.

### Safety evaluation of double-layered N-S1 PNp in vivo

**Fig. 10:**
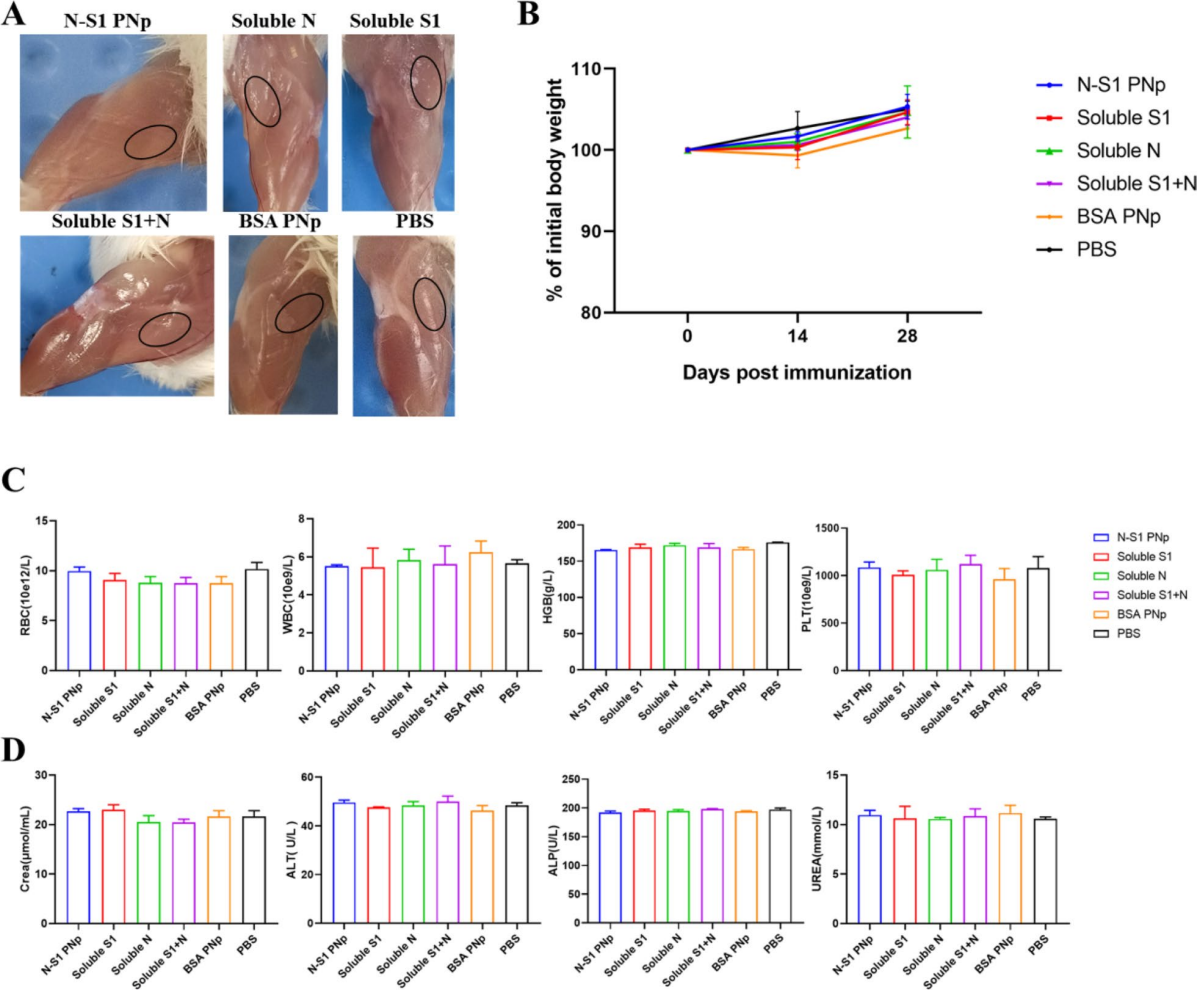
Safety of double-layer N-S1 PNp in mice. **A** images of adverse reactions at the injection site. **B** Body weight Changes of mice after immunization. **C, D** The whole blood cell count parameters (**C**) and biochemical indexes (**D**) of mice on the 14th day after immunization

To assess the safety of the double-layered N-S1 PNp in mice, the physical injury of the injection site, and body weight change were recorded, as well as the acute toxic effects were evaluated after immunization. No obvious detrimental physical consequences were observed at the injection sites after immunizations (Fig. 10A), and the weight of the mice gradually increased, with no statistically significant differences between the groups (Fig. 10B). A complete blood count (Fig. 10C) and serum chemistry panel (Fig. 10D) analysis showed no significant increase in alanine aminotransferase (ALT), urea nitrogen (UREA), alkaline phosphatase (ALP) and creatinine (Crea) levels after 14 days of immunization. There was no significant change in complete blood count index or liver and kidney function among the groups.

## Discussion

Developed SARS-CoV-2 vaccines face threats from the emergence and rapid spread of viral variants, it induced immunity by its prototype S protein, which exhibited wane over time, thus decreasing protection against COVID-19 [42–44]. In response, developing next-generation vaccines that aim to broaden the antigenic diversity of SARS-CoV-2 meanwhile generate diverse vaccine strategies for maintaining long-term high antibody levels is imperative. An ideal vaccine should induce broadly protective humoral and cellular immune responses against a diverse range of SARS-CoV-2 and its emerging variants, while conferring effective long-term protection for the elderly or immunocompromised individuals. To realize the ideal vaccine, it is necessary to refine immunogen design, utilize nanotechnology-based formulation, and implement a controlled release.

Nanotechnology is conducive to the development of new generations of SARS-CoV-2 vaccines. Since virions are nanoscale micro-organisms, designing immunogens with the same nanoscale, such as nanoparticles, is an ideal approach to stimulate the best immune efficacy [45]. Physiologically activated disassembling nanoparticles, such as the double-layer nanoparticle, boost innate immunity and trigger strong cellular and humoral immune responses with minimal cytotoxicity [46]. Double-layer nanoparticles have been demonstrated as a safe and effective strategy for integrating different antigen proteins or peptides as vaccines display to the immune system [31]. For example, self-disassembling double-layered M2e-NA protein nanoparticles and NP-peptide/4M2e-layered polypeptide nanoparticles could induced high-level specific long-lasting immunity that was against different influenza subtypes [33, 47]. To our knowledge, the almost pure double-layered protein nanoparticles contain high antigen loads, which comprised only antigenic proteins of interest. Repeated and orderly display of antigen on double-layered protein nanoparticles has been proved to promote an expanded germinal center reaction, resulting in increased B cell receptor mutation and plasma cell differentiation [48, 49]. At the same time, the high antigen loads of double-layered protein nanoparticles may exceed a threshold, which can overcome the neutralization resistance to virus variants with different antigenicity. It could facilitate the development of cross-neutralized SARS-CoV-2 vaccines. In addition, this design exhibits excellent stability against osmotic stress or changes in salt concentration, and lacks self-assembling structures or nanocarriers, like polymers, ferritin, or hepatitis B core protein, which can avoid off-target immune responses [50, 51]. Double-layer nanoparticles are easy to generate without special production systems and offer an effective substitute to traditional large-scale antigen manufacture or technology platforms. Moreover, the ethanol used for desolvation is relatively non-toxic, ensuring the safety of the produced nanoparticles. Overall, the fabrication process of double-layered protein nanoparticles is cost-effective, convenient, highly secure, and adaptable.

The evidence indicates that SARS-CoV-2 variants can effectively escape humoral immunity while exhibiting less capacity for evading T cells induced by vaccinations [52]. So, additional immunogenic structural proteins, such as nucleocapsid (N) protein, can be supplemented in combination with S protein to enhance immunogenicity and effective T-cell responses. Most convalescent patients (82%) mount detectable specific antibodies response against the N and S protein after SARS-CoV-2 infection [53]. Meanwhile, robust T cell responses, involving CD4^+^ and CD8^+^ cells, capable of recognizing multiple regions within N protein have also been found [17]. Vaccines for SARS-CoV-2 nucleocapsid have been developed based on different platforms. For example, based on the Ad5 vector N protein vaccine (Ad5-N), N protein subunit vaccines, nucleoside-modified along with lipid-nanoparticle (LNP) mRNA vaccine (mRNA-N). They were highly immunogenic, eliciting robust N-specific binding antibody and T-cell responses, but weak neutralizing antibody responses [54]. These findings have indicated that the N protein has potential value in augmenting vaccine-mediated protective immunity [55]. Thus, we considered incorporating S and N proteins into the design of the SARS-CoV-2 nanoparticle vaccine to overcome these weaknesses and stimulate strong protective B cell and T cell immune responses.

We fabricated the double-layered N-S1 protein nanoparticle with a core of conserved N protein containing a full-length nucleocapsid sequence, while a coating of S1 protein that includes RBD and its connected subdomain 1/2 ( SD1/SD2) sequences. A study has revealed regions of S without amino acid changes in SARS-CoV-2 through a bioinformatics approach [56]. Another study identified neutralizing antibodies against the SD1/SD2 displayed cross-reaction and protection in vivo, as SD1/SD2 is conserved [56, 57]. The clinical applications have demonstrated the potential of SD1/SD2 in developing a protective cross-neutralizing vaccine. Bispecific antibodies comprising SD1/SD2 and RBD exhibit remarkable efficacy in treating SARS-COV-2-infected mouse models [56].

Conservative N nanoparticle core (N PNp) was generated by ethanol desolvation, followed by cross-linking of the described above S1 onto the core particle surface by primary amine using DTSSP to fabricate a double-layered N-S1 PNp with a size of around 220 nm. Protein nanoparticles could increase the amount of antigen quickly taken up by APCs in vitro, thereby enhancing the effectiveness of subunit vaccines [58, 59]. In this study, N-S1 PNp is more efficiently taken up by phagocytes (macrophages and dendritic cells) than soluble protein antigens, promoting the maturation of BMDC in vitro, a signal that can trigger a robust immune response, which is relating to the size of N-S1 PNp. In the current study, nanoparticles or soluble antigens with a diameter of less than 10 nm flow rapidly through lymphatic organs, thus reducing the opportunity for APCs uptake [60]. Nanoparticles of around 200 nm were effectively directed to the lymph node and spleen, prolonging lymphatic organs' residence.

Our data showed that N-S1 PNp induced sustained humoral immunity and a strong T-cell immune synergistic response. The synergistic activity of N-S1 PNp stimulates high levels of antibodies that effectively neutralize SARS-CoV-2 spike pseudovirus, including prototype (WT), Delta, Omicron, and Omicron sub-variant lineages (BA.1, BA.2, BA.5, and BQ.1.1). In other words, N-S1 PNp can elicit cross-neutralization antibody responses. A previous study showed that the intense levels of neutralizing antibodies in vaccinated individuals provide advantages for vaccine efficacy and durability, as neutralizing antibodies can intercept and neutralize pathogens and trigger destructive innate immune responses [61]. The durability of the antibody response induced by N-S1 PNp is related to the characteristics of the crosslinking agent DTSSP which enabled protein nanoparticles gradual disassemble and antigenic proteins release in the APCs intracellular redox environment, promoting the acquisition of higher affinity and broader long-term immunity [62]. The SARS-CoV-2 infection involves both CD4^+^ and CD8^+^ T cell responses against spike protein, and sufficient CD4^+^ Tfh cell responses are required to induce high-level antibody responses in vivo [16, 17, 63]. We found that N-S1 PNp immunization induced robust CD4^+^ and CD8^+^ T cell responses in mice. The regulation of vaccine immune response is triggered by Toll-like receptors (TLRs) on DCs through the downstream NF-κB signaling pathway [64, 65]. Mature DCs express MHC molecules as well as co-stimulatory molecules bind to TCR and activate T cells [66, 67], which differentiate into T follicular helper cells (Tfh), regulatory T cells (Tregs), and cytotoxic or “killer” T cells (Tc) [68]. Then naive B cells undergo differentiation into plasma cells and memory B cells, ultimately contributing to the formation of GCs [34]. Durable high-titer neutralizing antibodies rely on the robust and persistent Tfh cell response and GCs response triggered by the vaccine, thereby conferring protection against SARS-CoV-2 infection [40, 41, 69]. Therefore, vigorously promoting Tfh and GCs cell responses is crucial for developing a new generation of SARS-CoV-2 vaccines. Results indicate that N-S1 PNp vaccination can promote Tfh cell differentiation, inducing skewed Tfh cells toward CXCR5^+^ PD-1^+^ Tfh differentiation, which is conducive to stimulating broad and specific NAb responses. Given the critical role of Tfh cells in assisting B cells in GCs, as expected that N-S1 PNp would also elicit a robust GCs B cell response. Overall, N-S1 PNp immunization induces high-level T-cell responses to promote the production of neutralizing antibodies that are cross-reactive for a long time.

## Conclusions

In summary, these findings demonstrate that N-S1 PNp induces strong and long-lasting specific humoral and cellular responses against SARS-CoV-2, which may confer broad efficacy against future new variants and warrant further development. This study provides a new idea for the design of next-generation SARS-CoV-2 vaccines based on the B and T cells response coordination. Finally, the affordability of N-S1 PNp will facilitate the resolution of low-income countries' challenges in accessing safe and effective SARS-CoV-2 vaccines to contain the COVID-19 pandemic.

## Methods

### Animals, cells, and peptides

Female BALB/c mice aged 6–8 weeks were procured from Henan Sikebesi Biological Technology Co. Ltd (Henan, China) and housed in a specific pathogen-free (SPF) animal facility after approval by the Laboratory Animal Ethics Review Committee.

*E.coli.* DH5α and BL21 (DE3) Chemically Competent Cells were purchased from TransGen Biotech Co., Ltd. (Beijing, China). Human embryonic kidney 293F (HEK293F) cells, cultured in SMM 293-TII Expression Medium (Sino Biological; M293TII), were preserved by Henan Provincial Key Laboratory of Animal Immunology at Henan Academy of Agricultural Sciences (Henan, China). DC2.4 and RAW264.7 cells were obtained from ATCC (Manassas, VA, USA).

14 overlapped synthetic peptides pool of the SARS-CoV-2 N protein was used for ELISpot analysis, with synthesis by GL Biochem (Shanghai, China), as shown in Additional file 1: Table S2.

### Design, expression, and purification of S1 protein

The S1 protein gene sequence was retrieved from NCBI (Spike residues 319–668, GenBank: MN908947.3). The signal peptide encoding sequence of the SARS-CoV-2 spike protein was employed in the S1 protein constructs to facilitate secretion. The S1 protein plasmid was synthesized by Sangon Biotech (Shanghai, China). The products were ligated into the pcDNA3.1 (+) vector and subsequently introduced into *E.coli.* DH5α competent cells through transformation. The recombinants were amplified and abstracted utilizing the EndoFree Plasmid Maxi Kit (CWBIO; CW2104). The plasmid was transiently transfected into HEK293F using PEI (Yeasen Biotechnology, China) [70]. The cell supernatant was collected and filtered to identify protein 72h after transfection. Then proteins were purified by using an immunoaffinity chromatography column based on the SARS-CoV-2 Spike protein-specific mAb preserved in our laboratory. The eluted fractions were pooled and exchanged to 0.01M PBS pH 7.4 via Sephadex G-25 (GE Healthcare, USA).

### Design, expression, and purification of N protein

Based on the original SARS-CoV-2, we obtained the full-length sequence of N protein (GenBank: NC045512.2) from NCBI. The N protein recombinant plasmid was modified with a secretory expression signal peptide sequence, adding a six-histidine-tag sequence to the C terminal of the protein. The modified plasmid was inserted into the pET28a vector and transformed into competent BL21 (DE3) *E. coli*, obtaining a single colony. Then PCR identification of the recombinant BL21 strain was cultured into a 10 mL LB liquid medium containing 50 μg/mL kanamycin for 12 h. The pre-culture was diluted 1:100 into 1 L LB medium for 3 h. After adding Isopropyl β-D-1-thiogalactopyranoside (IPTG) with a final concentration of 0.2 mM, continuing to induce for 12 h at 16 °C. Bacterial cultures were harvested by centrifugation and sonicated from collected supernatant, and then purified through binding into Ni Sepharose™ Excel affinity column (His Trap ™ Excel, GE) [21].

### Fabrication of protein nanoparticles

The N core PNp was formed by desolvating with absolute ethanol, and the N-S1 protein double-layered nanoparticle was prepared through crosslinking reactions. Specifically, a four-fold volume of absolute ethanol was slowly dripped at 1 mL/min into N protein solution while constant stirring at 600 rpm for 1 h, obtaining the PNps by discarding the supernatant after centrifugation at room temperature and 15,000 ×*g* for 15 min. The PNp was sufficiently resuspended in a solution of S1 protein by sonication. Then 5 mM DTSSP (Sigma-Aldrich; 803,200) was added and continuously stirred for 1.5 h at 4 °C before being quenched with 30 mM Tris–HCl solution (pH 7.4) and stirred for an additional 15 min, centrifuged at 15 000 rpm for 30 min to discard the supernatant, resuspended the remaining in 0.5 mL PBS, stabilized by sonication on an ice bath. The fabrication of BSA double-layered PNp was analogous to the above-described method.

### Nanoparticle characterization

The nanoparticle size of the N-S1 protein double-layered nanoparticle and the N core PNp were assessed by dynamic light scattering (DLS) analysis. The samples in the quartz cuvette were incubated for 3 min in the Malvern Zetasizer Nano ZS90 (Malvern, Worcestershire, UK), and measurements were taken at 25℃ with 10 scans for 30 s each. The composition of the N-S1 protein double-layered nanoparticle was characterized by 12.5% and 7.5% SDS-PAGE, followed by Coomassie blue staining and Western blotting. Anti-his-tag monoclonal antibody (Proteintech; 66,005–1-Ig) and anti-S1 mouse mab (prepared and stored in our laboratory and developed according to standard procedures) were used to analyze the nanoparticles by Western blotting [71].Then the intensity of S1 protein bands was analyzed using Image J software. The protein concentration of the N-S1 protein double-layered nanoparticle solution was determined using the BCA kit (Beyotime; P0012). A droplet of nanoparticle solution was applied onto a 100 mesh carbon-coated TEM copper grid (Beijing Zhongjingkeyi Technology Co., Ltd., China) and air-dried for 2 min. Subsequently, the excess nanoparticle solution was removed from the side of the TEM grid using filter paper, followed by immediate incubation with a drop of 3% phosphotungstic acid negative staining solution for 2 min on the same grid. Before the grid was subjected to air drying at room temperature for 4 h, preceded by another round of wicking from its side with filter paper. TEM imaging with a Thermo Scientific™ Talos™ L120C transmission electron microscope at 120 kV.

The surface plasmon resonance (SPR) assays were conducted using the CM5 sensor Chip (Cytiva; 29,149,604). The binding affinity difference of human ACE2 protein to soluble S1 protein or N-S1 PNp was determined using a Biacore X100 device (GE Healthcare, USA) with the HBS-EP running buffer (Cytiva; BR100669). The human ACE2-His protein was diluted to a concentration of 30 μg/mL in pH 4.0 10 mM sodium acetate buffer for covalent coupling to immobilization. The soluble S1 protein or N-S1 PNp was diluted to concentrations ranging from 15.625 nM to 500 nM in HBS-EP buffer and flowed over the chip surface, respectively. Following an association period of 90 s, a dissociation period of 90 s ensued, then a final regeneration step with glycine pH 1.5 regeneration buffer. The binding affinity value (K_D_) was determined.

### Isolation and maturation of dendritic cells in bone marrow (BMDCs) assay

To obtain the BMDCs, bone marrow hematopoietic stem cells were isolated from the femur of C57BL/6 mice. Bone marrow cell suspensions were prepared in RPMI Medium 1640 (Solarbio; 31,800) with 10% FBS through a 70 μm cell strainer. The cells were then differentiated into BMDCs by the addition of GM-CSF (20 ng/mL; MCE; HY-P7361A) and IL-4 (10 ng/mL; Solarbio; P00196), followed by incubation at 37 °C with 5% CO2. After 7 days of culture, loosely adherent and floating cells were harvested for subsequent experiments.

BMDCs were directly plated with 1 × 10^6^ per well into the 24-well plate, followed by stimulation using soluble protein (N or S1) or double-layered N-S1 PNps 5 μg/mL for 24 h, which the corresponding amount of proteins in each group was the same. Moreover, the negative and positive wells were stimulated with PBS and LPS, respectively. Then, BMDCs were harvested and centrifuged at 400 ×*g* for 5 min, before being incubated with anti-CD11c-FITC, anti-CD86-PE, and anti-CD80-APC. Finally, cells were analyzed using FCM as described in Methods.

### In vitro cellular uptake assay

To assess the cellular uptake of double-layered N-S1 PNps by antigen-presenting cells (APCs), indirect immunofluorescence (IFA) was performed. Specifically, RAW264.7 and DC2.4 cells were seeded into 96-well plates for overnight culture at a density of 8 × 10^4^ cells/well. The cells were incubated with 15 μg/mL soluble N protein, 35 μg/mL soluble S1 protein, 50 μg/mL N-S1 PNps, a protein mixture of 35 µg S1 and 15 μg N, or PBS as a negative control for 2 h. After removing the medium, the cells were fixed with pre-cooled methanol for 10 min and then washed with PBS three times. The cells were blocked with 5% skim milk in PBST (PBS containing 0.05% Tween-20) for 2 h at 37 ℃. Next, the cells were incubated with mouse-specific S1 and N monoclonal antibodies against SARS-CoV-2, respectively, then labeled with the Alexa Fluor488-conjugated goat-anti-mouse secondary antibody (Solarbio; A0428). After 1 h incubation, DAPI (Solarbio; C0065) was used to stain the cells and incubated for 4 min at room temperature in the dark. The cells were washed three times with PBS before imaging by fluorescence microscopy (Zeiss, Germany).

### In vitro cytotoxicity assay

To evaluate the cytotoxicity of double-layered N-S1 PNps, different concentrations of double-layered N-S1 PNps were incubated with RAW264.7 and DC2.4 cells for 24 h. They were plated into 96-well plates with a density of 1 × 10^4^ cells/well 12 h in advance. After incubation, cytotoxicity was evaluated by Cell Counting Kit-8 (Dakewe; 6,073,213). Briefly, the cell plate was added with DMEM medium containing 10% CCK-8 reagent and cultured for 4 h before measuring the absorbance of OD 450 nm.

### Immunization in mice study designs

Female mice (BALB/c strain, 6–8 weeks, n = 8) were randomly allocated to six groups and immunized intramuscularly with two doses of 100 μL vaccines in PBS containing aluminum hydroxide (Croda) and CPG 1018 (PARR BIO) adjuvants, spaced 21 days apart. The mice received the following vaccines respectively: 10 μg N-S1 protein double-layered nanoparticle, 7 µg soluble S1 protein, 3 μg soluble N protein, 10 μg BSA double-layered protein nanoparticles, or a formulation comprising a protein mixture of 7 µg soluble S1 protein and 3 μg soluble N protein (a dose of 10 µg total soluble protein). A placebo group was recognized as a non-immunized control, which received 100 μL of PBS. The vaccine mixtures were injected into the hind leg gastrocnemius muscle. Since the initial immunization, blood samples were gathered 1 day before prime immunization, and weekly after immunization for titer measurement. Additionally, immune sera were collected bi-monthly following the boost immunization to analyze its impact on long-term immunity.

### ELISA binding antibodies assay

Serum samples from various groups of mice were analyzed using standard indirect Enzyme-linked immunosorbent assay (ELISA) to assess the IgG and its isotype-specific antibody levels of S1 or N proteins. The purified S1 protein (WT, Beta, Delta, Lambda, and Omicron) was coated into ELISA plates by overnight incubation at 4 ℃ for subsequent binding assay. The next day, each well was blocked with 5% skim milk in PBST (PBS with 0.05% Tween-20) for 2 h at 37 ℃. Then, the serum was performed as a primary antibody and serially diluted with ELISA-blocking buffer was incubated for 1 h at 37 ℃. The S1-specific IgG, IgG1, and IgG2a antibodies were titrated by incubating with the horseradish peroxidase (HRP)‐conjugated Goat Anti-Mouse IgG (Promega; W4021), IgG1 or IgG2a (Jackson ImmunoResearch Laboratories; 115–035-205, 115–035-206) as detection antibodies for 1 h at 37 ℃. Each well was developed with TMB Single-Component Substrate solution (Solarbio; PR1200) in the dark for 10 min. 2 M H_2_SO_4_ was used to stop color development. Finally, the absorbance at 450 nm was performed immediately using a multi-functional enzyme standard instrument (Omega, Germany). Similar to the Method described above, indirect ELISA was employed to ascertain the endpoint titers of N-protein-specific IgG, IgG2a, and IgG1.

### Binding kinetics of antigen-specific IgGs through SPR assays

Binding kinetics of N-S1 PNps vaccinated-murine S1-specific serum IgG was determined using a Biacore X100 equipment (GE Healthcare, USA) and Amine coupling kit (GE Healthcare; BR-1000–50) with the Series S CM5 sensor Chip (Cytiva; 29,149,604). After injecting a mixture of EDC/NHS (1:1) to activate the CM5 Sensor Chip, Anti-Mouse antibodies (Cytiva; BR100838) were diluted to 30 μg/mL in 10 mM sodium acetate buffer pH 5.0 for immobilization using HBS-EP running buffer (Cytiva; BR100826). Residual-free NHS-esters were rendered inactive by the injection of 1 M ethanolamine-HCl, pH 8.5. Sera at 21 days post-boost immunization were diluted 1:100 in 1 × HBS-EP buffer and applied to the active flow cell for capture by the immobilized antibody for 60 s. S1 proteins of SARS-CoV-2 WT, beta, delta, lambda, and omicron were diluted with concentrations ranging from 0 to 25 nM in 1 × HBS-EP (pH, 7.4) buffer, respectively, which were applied a multi-cycle kinetic method for analyzing the binding of captured murine IgG antibodies. Finally, Glycine–HCl of pH 1.7 was used for the regeneration step. Binding kinetics were determined with a global kinetic fit model (1: 1 Langmuir, Biacore X100 evaluation software, Cytiva) and showed by K_D_ values.

### ACE2-receptor-blocking antibodies assay

ACE2-receptor-blocking antibodies were analyzed using ELISA. Ninety-six-well ELISA plates were coated with S1 protein overnight incubated at 4 °C. The serum obtained from immunized mice was serially diluted and incubated at 37 °C for 1 h. PBS was added into wells as a control simultaneously. After 1 h, histidine-tagged hACE2 was introduced to plates and left at 37 °C for another hour, followed by washing three times by PBST (PBS with 0.05% Tween-20). HRP-conjugated mouse anti-histidine IgG (Proteintech; HRP-66005) was used as the secondary antibody, incubated for 1 h at 37 °C. The inhibition rate was determined using the following equation: [(OD value of control—OD value of samples/OD value of control) × 100%]. The titer was calculated using GraphPad Prism 8.0.2 based on the serum dilution that caused a 50% inhibition of receptor binding.

### Pseudovirus neutralization assay

A recombinant replication-deficient vesicular stomatitis virus (VSV) backbone, in which the G gene was replaced with the firefly luciferase (Fluc) reporter gene (G*ΔG-VSV), was pseudotyped with the SARS-CoV-2 Prototype, Delta, Omicron, and Omicron sub-variant lineages (BA.1, BA.2, BA.5, and BQ.1.1) S protein according to published pseudotyping protocols [72–74]. Huh-7 cells with stable expression of hACE2 (Huh-7/hACE2) and sufficient pseudotyped virus suspension were prepared in advance. Mouse serum samples were subjected to serial dilutions and pre-incubated with equal volumes of different SARS-CoV-2 pseudoviruses suspension including the Prototype, Delta, Omicron, and Omicron sub-variant lineages (BA.1, BA.2, BA.5, and BQ.1.1) (Beijing Tiantan Pharmaceutical Biotechnology Development Company, China) in 96 wells plates for 1 h at 37 °C before transferring the mix to Huh-7/hACE2 cells. Inoculated Huh-7/hACE2 cells were incubated for another 24 h at 37 °C to express the reporter gene luciferase. In each plate, the cell control (CC) containing only cells and the virus control (VC) comprising virus as well as cells were established. Finally, the supernatant was removed and discarded, and 100 µL lysis reagent with luciferase substrate (Beyotime, China) was added to each well. A multi-mode microplate reader (Molecular Devices, USA) was used to analyze the relative light unit (RLU). The inhibition rates of infection for each dilution of the sample were determined based on the RLU values as follows: inhibition rate = [1–(average RLU of sample–average RLU of CC)/(average RLU of VC–average RLU of CC)] × 100%. The 50% pseudovirus neutralizing titers (pVNT50) were determined as the reciprocal of the highest serum dilution that caused 50% inhibitory compared to the average of the virus control wells.

### Mouse splenocyte isolation, IFN-γ, and IL-4 enzyme-linked immunospot assay

On day 21st after the boost immunization, splenocytes of vaccinated mice were collected from all immunization groups as single-cell suspensions using the mouse spleen lymphocyte isolation kit (Solarbio; P8860). Freshly isolated splenocytes were plated directly at a density of 4 × 10^5^ per well in RPMI Medium 1640 (Solarbio; 31,800) supplemented with 10% FBS for restimulation. Splenocyte of each well was stimulated with the N peptide pool (consisting of equal amounts of 14 overlapping amino acids synthetic peptides of SARS-CoV-2 N protein, at a final concentration of 8 µg/mL per peptide), purified S1 protein (A final concentration of 14 µg/mL), or mock-restimulation in wells pre-coated with anti-IFN-γ or anti-IL-4 antibodies for corresponding lymphocyte ex vivo stimulation in a volume of 20 µL/well. Incubated at 37 °C in a 5% CO2. Each stimulation condition was employed in triplicate wells. After 36 h, AEC substrate solutions (Dakewe; 2,210,002 and 2,210,402) were added to the plates for 20 min, then thoroughly rinsed with purified water and subsequently air-dried prior to counting. The spots were enumerated and analyzed with an ELISpot plate reader (ImmunoSpot^®^ S6 Ultra Analyzer [CTL], US). Spot numbers were assessed and normalized to spots/10^6^ splenocytes.

### Cytokine secretion levels measurement assay

Lymphocytes were separated from spleens and re-stimulated with a synthetic N peptides pool or purified S1 protein as described in Methods. After 60 h, concentrations of IFN-γ, IL-2, and IL-4 in the splenocyte culture supernatants (1 × 10^6^ cells/mL) were determined using commercial Mouse IFN-γ ELISA KIT (Solarbio; SEKM-0031), Mouse IL-4 ELISA KIT (Solarbio; SEKM-0005), Mouse IL-2 ELISA KIT (Solarbio; SEKM-0004) according to the instructions for uses, respectively. Signals were measured at 450 nm and 630 nm wavelengths within 5 min by the multi-functional enzyme standard instrument (Omega, Germany).

### Lymphocyte proliferative activity assay

Lymphocytes were isolated and re-stimulated from immunized mice 3 weeks after the final immunization as described in Methods. The splenocytes (1 × 10^6^ cells/mL) were prepared and stimulated for 60 h at 37 °C. Moreover, the negative wells were treated with the medium as a stimulant. The proliferation of lymphocytes was assessed with Cell Counting Kit-8 (CCK-8) (Dakewe; 6,073,213). The cell proliferation rate was determined using the stimulation index (SI). SI = (the mean of OD 450 nm values of the N peptide pool or S1 protein stimulated wells)/(the mean of OD 450 nm values of negative wells).

### Flow cytometry assay

Spleens of mice were excised in 3 weeks after the boost vaccination and splenocytes suspension (1 × 10^6^ cells/mL) was prepared as described in Methods. Red blood cells were first lysed with ACK Lysis Buffer (Beyotime; C3702) before cell surface markers were stained using various antibodies. Then, the lymphocytes were stained and incubated with anti-CD3-FITC, anti-CD4-PerCP-Cy5.5, and anti-CD8-APC in the dark for 25 min at 4 °C. To evaluate GCs formation in spleens, Tfh cells were stained with anti-CD4-PerCP-Cy5.5, anti-CXCR5-BV421, anti-ICOS-APC, and anti-PD-1-PE. In addition, GCs B cells were stained with anti-B220-BV510, anti-CD95-APC, and anti-GL7-Alexa Fluor 647. To assess the maturation of DCs, the cells were stained with anti-CD11c-FITC, anti-MHC class II-BV421, and anti-CD80-APC. To measure the percentage of central memory of T cells, the cells were stained with anti-CD3-FITC, anti-CD62L-PerCP-Cy5.5. All fluorochrome-conjugated antibodies were purchased from Biolegend (BioLegend, USA). Moreover, stained cells were washed and centrifuged twice (at 300 ×*g* for 5 min each time) before flow cytometry analysis. The resuspended cells were detected utilizing the CytoFLEX (Beckman Coulter, USA) and the acquired data were analyzed with FlowJo software (BD Biosciences).

### Reverse-transcription quantitative polymerase chain reaction (RT-qPCR) assay

The spleen lymphocytes of mice in different immunization groups were harvested, then extracted the total RNA by the RNAiso Plus (Takara; 9108) method and reverse-transcripted into cDNA utilizing PrimeScript™ RT Master Mix (Takara; RR036A). The qRT-PCR was performed using the ChamQ Universal SYBR qPCR Master Mix (Vazyme Biotech; Q711-02) and Applied Biosystems™ 7500 Fast Real-Time PCR System (Thermofisher, USA). Primer sequences utilized are shown in Additional file 1: Table S1, while the relative gene expression values were determined through the 2^−ΔΔCt^ method with β-actin as control.

### H&E and immunofluorescence staining assay

Briefly, inguinal lymph node samples were harvested from immunized mice 3 weeks after boost immunization, fixed in formalin, and embedded in paraffin (FFPE). Then paraffin-embedded tissue blocks were sectioned and incubated with hematoxylin for 5 min. The sections were subjected to a 30-s treatment with 1% hydrochloric acid alcohol before further treatment with eosin for 2 min. The results were viewed through an orthotopic optical microscope (Nikon Eclipse CI, Japan).

The immunofluorescence staining was performed on four-micron sections following standard procedures, which included deparaffinization, antigen retrieval, circle of the spontaneous fluorescence quenching, BSA blocking, primary and secondary antibody incubation, as well as counterstaining with DAPI. The primary antibody was the Rabbit Anti-CD45 antibody (Bioss; bs-4819R). Finally, sample sections were viewed on an upright fluorescence microscope (Nikon Eclipse Ci, Japan).

### RNA-Seq assay

Total RNA extracted from 15 to 30 μg spleen tissue on day 21 post-boost immunization. The quality of RNA was inspected using a NanoDrop One spectrophotometer (NanoDrop Technologies, Wilmington, DE), Qubit 3.0 Fluorometer (Life Technologies, Carlsbad, CA, USA), and agarose gel electrophoresis. The RNA sample preparations were conducted with 2 μg of total RNA per sample as input material. mRNA was enriched using magnetic beads before synthesizing double-stranded cDNA to generate Sequencing libraries. Next, the procedures of terminal repair, A-tailing, and adapter added were implemented, and following fragment screening and PCR, obtained the final library, which was generated using MGIEasy RNA Library Prep Kit for BGI^®^. Finally, the libraries were sequenced using a BGI DNBSEQ platform (BGI Genomics, Shenzhen, China) provided by Wuhan Benagen Technology Co., Ltd.

Adaptors and low-quality sequences that negatively impact subsequent assembly were filtered out from the raw data by FastQC (v0.11.9, http://www.bioinformatics.babraham.ac.uk/ projects/fastqc/). Then, the trimmed clean reads were mapped to the Mus musculus reference genome (http://ftp.ensembl.org/pub/current_fasta/mus_musculus/dna/Mus_musculus.GRCm39.dna.toplevel.fa.gz) using the Star aligner (v2.7.9a, https://github.com/alexdobin/STAR). To compare the level of similarity among the samples, sample correlation analysis was conducted (Additional file 1: Fig. S1A). Differential expression genes (DEG) were performed by DESeq2 (v1.34.0, http://www.bioconductor.org/packages/release/bioc/html/DESeq2.html). The DEGs refer to a significant false discovery rate-adjusted P value (FDR) < 0.05 based on three biological replicates. Gene Ontology (GO) and Kyoto Encyclopedia of Genes and Genomes (KEGG) enrichment analyses for the DEGs using the clusterProfiler version 3.14.3 (http://www.bioconductor.org/packages/release/bioc/html/clusterProfiler.html). In addition, the pathways with a P-value < 0.05 were selected and the top 20 are shown.

### In vivo safety and serum chemistry

BALB/c mice were intramuscularly injected with 20 μg of double-layer N-S1PNp, while soluble S1 or N proteins, double-layer BSA PNp, and PBS control groups were set up. Record the limb injuries and weight changes at the injection site after immunization, and collect blood on the 14th day for testing the whole blood cell count parameters (WBC, RBC, PLT, and HGB levels) and biochemical indexes (Crea, ALT, URA, and ALP levels) to evaluate acute toxic effects.

### Statistical analysis

All figures were prepared with GraphPad Prism 8.0 and FlowJo 10.6.2 Software. The P values were obtained through one-way ANOVA analysis followed by multiple comparisons post hoc tests using the IBM SPSS Statistics 21.0 for group comparisons. Differences with a P value of < 0.05 were considered significant: ***p-value < 0.001, **p-value < 0.01, *p-value < 0.05.

### Supplementary Information

**Additional file 1: Figure S1.** Transcriptome quality assessment and DEGs in spleens. **Figure S2.** GO and KEGG enrichment analysis of DEGs. **Table S1.** Primer sequences. **Table S2.** Amino acid sequences of 14 overlapped synthetic peptides of SARS-CoV-2 N protein.

## Data Availability

The datasets used and/or analyzed during the current study are available from the corresponding author on reasonable request.
