# Supplementary material for: Double-layered N-S1 protein nanoparticle immunization elicits robust cellular immune and broad antibody responses against SARS-CoV-2
Source: J Nanobiotechnology. 2024 Jan 30;22:44. doi: 10.1186/s12951-024-02293-y (PMC10825999; doi:10.1186/s12951-024-02293-y)
Supplement: Supplementary file 1 — Additional file 1: Figure S1. Transcriptome quality assessment and DEGs in spleens. Figure S2. GO and KEGG enrichment analysis of DEGs. Table S1. Primer sequences. Table S2. Amino acid sequences of 14 overlapped synthetic peptides of SARS-CoV-2 N protein. [file 12951_2024_2293_MOESM1_ESM.docx]

**Double-layered N-S1 protein nanoparticle immunization elicits robust cellular immune and broad antibody responses against SARS-CoV-2**

Ruiqi Li^1, 2, 3, 4^, Zejie Chang^1, 4, 5^, Hongliang Liu^6^, Yanan Wang^5^, Minghui Li^5^, Yilan Chen^4^, Lu Fan^4^, Siqiao Wang^4, 5^, Xueke Sun^4, 5^, Siyuan Liu^4, 5^, Anchun Cheng^1^, Peiyang Ding^6*^ and Gaiping Zhang^1, 2, 3, 4, 5, 6*^

^1^ College of Veterinary Medicine, Sichuan Agricultural University, Chengdu, 611130, China.

^2^School of Advanced Agricultural Sciences, Peking University, Beijing, 100080, China.

^3^Longhu Laboratory, Zhengzhou, 450046, China

^4^Henan Provincial Key Laboratory of Animal Immunology, Henan Academy of Agricultural Sciences, Zhengzhou, 450002, China.

^5^College of Animal Medicine, Henan Agricultural University, Zhengzhou, 450046, China

^6^School of Life Sciences, Zhengzhou University, Zhengzhou, 450001, China.

^*^Correspondence: [zhanggaip@126.com](mailto:zhanggaip@126.com) and [dingpeiyang1990@163.com](mailto:dingpeiyang1990@163.com)


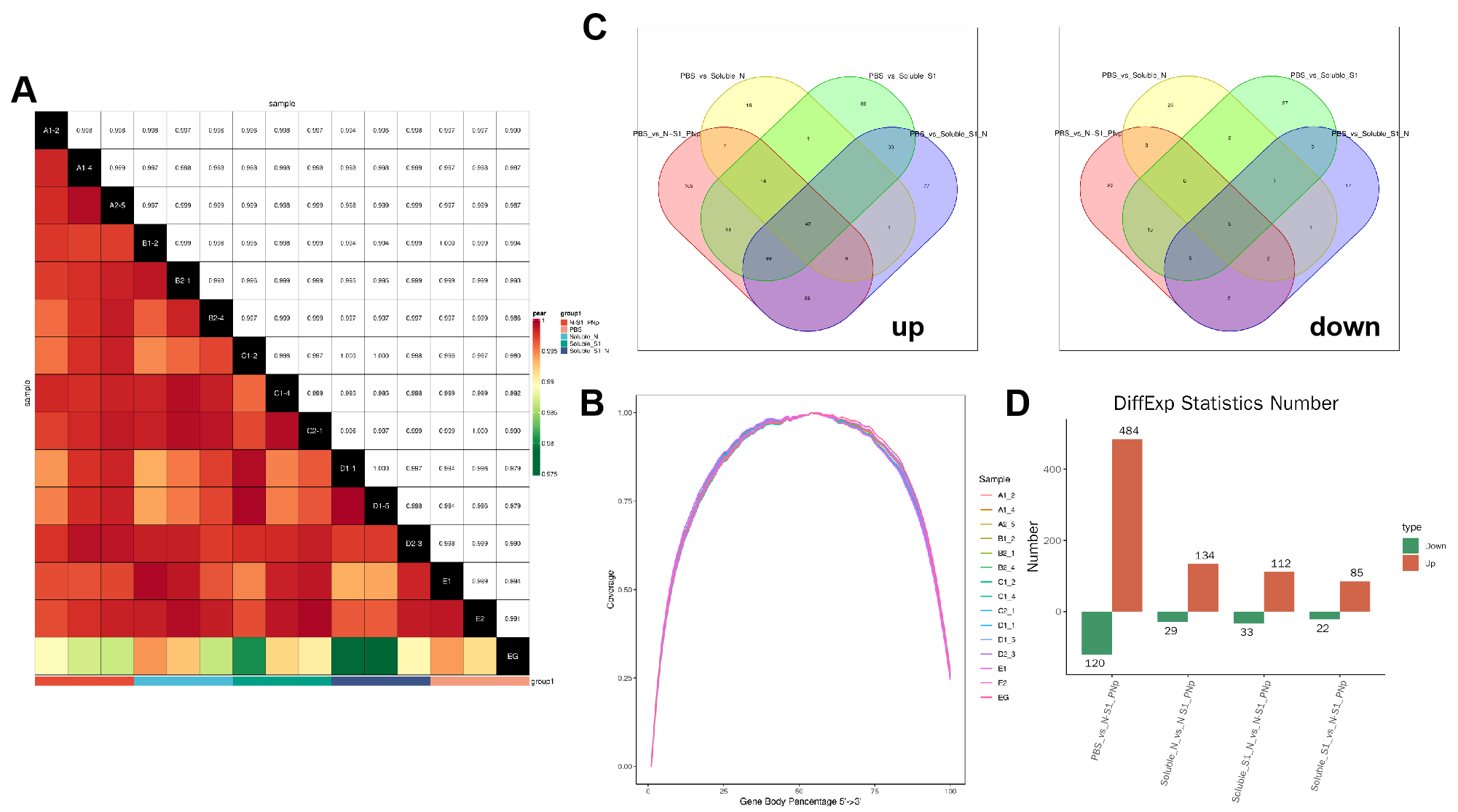


**Figure S1.** Transcriptome quality assessment and DEGs in spleens. **A** The heat map displays the Pearson correlation coefficient among samples included in the analysis. The intensity of the red color indicates a stronger correlation between the two samples. Different colors at the bottom of the heat map represent distinct groups, including the N-S1 PNp group (red), Soluble N (light blue), Soluble S1 (green), Soluble S1 + N (dark blue), and PBS control group (pink). **B** Sequencing coverage analysis, this curve reflects the coverage of sequencing reads on genes (along 5 'to 3'). A represents group N-S1 PNp, B represents group Soluble N, C represents group Soluble S1, D represents group Soluble S1+N, and E represents the PBS control group, with 3 biological replicates in each group. **C** Venn diagram of upregulated DEGs (left) and downregulated (right), with different colors representing different groups, while overlapping regions indicate shared genes between groups. **D** The bar graph displays the DEGS numbers, with upregulated genes indicated in red and downregulated genes indicated in green


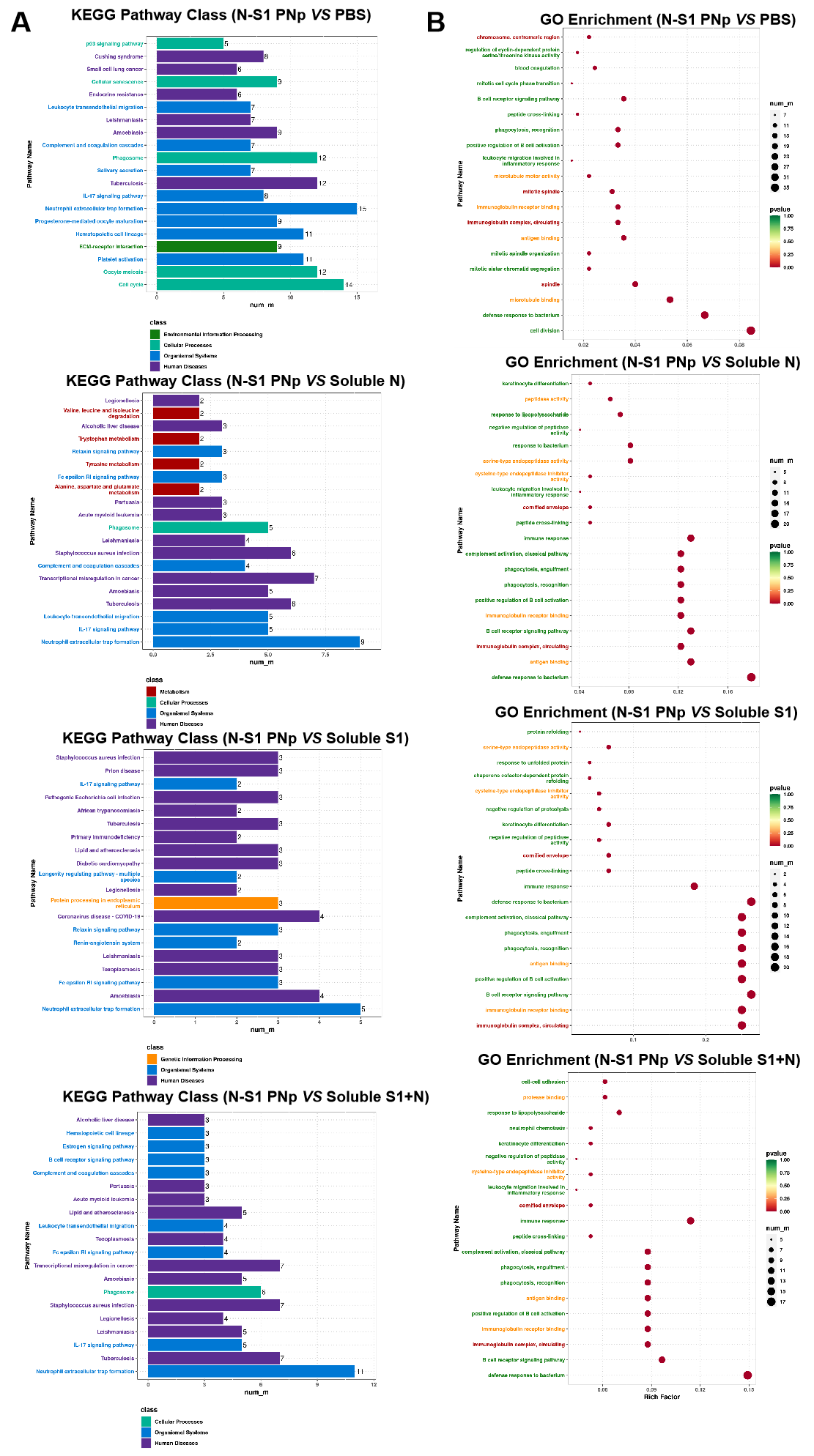


**Figure S2.** GO and KEGG enrichment analysis of DEGs. The KEGG enrichment analysis **(A)** and GO enrichment bubble plot **(B)** of N-S1 PNp versus PBS, soluble N, soluble S1, and soluble S1+N, ranking the top twenty annotations were listed based on correlation with each functional annotation. The vertical axis is the pathway name, the size of the circle or the horizontal axis represents the number of DEGs, the color represents the richness factor, and the color is the rich factor, representing p.adjust

**Table S1.** Primer sequences.

| Gene Name | Forward Primer (5’-3’) | Reverse Primer (5’-3’) |
| --- | --- | --- |
| IRF4 | GCCCAACAAGCTAGAAAG | TCTCTGAGGGTCTGGAAACT |
| IRF8 | GATCGAACAGATCGACAGCA | AAGCATCCACCTGATTG |
| Bcl6 | CCCTGTGAAATCTGTGGCACTC | ACACGCGGTATTGCACCTTG |
| Blimp1 | AGTAGTCAGTCGCTCGCTCA | CGGTCCCTCCTTTTCTACGG |
| AID | GGACAGCCTTCTGATGAAGC | GAGCAGGAGGTGGCACTATC |
| β-actin | AGAGGGAAATCGTGCGTGAC | CAATAGTGATGACCTGGCCGT |

**Table S2.** Amino acid sequences of 14 overlapped synthetic peptides of SARS-CoV-2 N protein.

| Peptide | Sequence |
| --- | --- |
| 1 | TQALPQRQKKQQTVTLLPAADLDDFSKQLQQSMSSADSTQA |
| 2 | ERSGARSKQRRPQGLPNNTASWFTALTQHGKEDLK |
| 3 | EAGLPYGANKDGIIWVATEGALNTPKDHIGTRNPA |
| 4 | MSGKGQQQQGQTVTKKSAAEASKKPRQKRTATKAY |
| 5 | VILLNKHIDAYKTFPPTEPKKDKKKKADETQALPQ |
| 6 | SDNGPQNQRNAPRITFGGPSDSTGSNQNGERSGAR |
| 7 | TSPARMAGNGGDAALALLLLDRLNQLESKMSGKGQ |
| 8 | IRQGTDYKHWPQIAQFAPSASAFFGMSRIGMEVTP |
| 9 | GKEDLKFPRGQGVPINTNSSPDDQIGYYRRATRRI |
| 10 | RATRRIRGGDGKMKDLSPRWYFYYLGTGPEAGLPY |
| 11 | GTRNPANNAAIVLQLPQGTTLPKGFYAEGSRGGSQ |
| 12 | SRGGSQASSRSSSRSRNSSRNSTPGSSRGTSPARM |
| 13 | TATKAYNVTQAFGRRGPEQTQGNFGDQELIRQGTD |
| 14 | GMEVTPSGTWLTYTGAIKLDDKDPNFKDQVILLNK |
